# A comprehensive review on structural, chemical, and functional perspectives of dietary polysaccharide-driven gut microbiota modulation: Mechanisms and challenges

**DOI:** 10.1016/j.bbrep.2026.102575

**Published:** 2026-04-04

**Authors:** Athira Jayasree Subhash, Mohamed Abdin, Gafar Babatunde Bamigbade, Maduni Paththuwe Arachchi, Naeem Ullah, Mutamed Ayyash

**Affiliations:** a Department of Food Science, College of Agriculture and Veterinary Medicine, United Arab Emirates University (UAEU), P.O. Box 15551, Al Ain, United Arab Emirates; b Agriculture Research Center, Food Technology Research Institute, Giza, 12611, Egypt

**Keywords:** Polysaccharides, Gut microbiota, Modulators, Health promotion, Metabolic regulation, Clinical trials

## Abstract

Polysaccharides in the diet have been increasingly recognized as major modulators of gut microbiota and multifunctional ingredients in food systems. Despite extensive research on various polysaccharides regarding their prebiotic activities, a comprehensive approach towards an integration of structural characteristics, chemical modifications, mechanistic degradation pathways, and food processing effects is still limited. This review aims to compile existing knowledge regarding the molecular characteristics of polysaccharides, including their monosaccharide composition, glycosidic linkages, branching, and molecular weight, in relation to their utilization by gut microbiota, production of short-chain fatty acids, and associated metabolic effects in the human body. Furthermore, this review will focus on carbohydrate-active enzymes and cross-feeding effects that play a major role in determining the biological activities of polysaccharides. The effects of food processing and food systems on polysaccharide functionality and utilization will also be considered. Despite promising therapeutic and nutritional potential, existing inconsistencies in research methods and a lack of standardized approaches in studying mechanistic effects are major hurdles in developing novel food systems that are targeted towards modulating gut microbiota.

## Highlights

• Polysaccharides modulate gut microbiota and enhance metabolic health.
• Structural diversity of polysaccharides drives selective microbial fermentation.
• Native and modified polysaccharides act as promising next-generation prebiotics.
• Future research should integrate multi-omics and clinical validation approaches.

## 1 Introduction

The human gastrointestinal (GI) tract microbiota, an intricate network of microorganisms, is essential for the maintenance of host health. These microorganisms are involved in the metabolism of nutrients, modulation of the host's immune response, and protection against pathogenic microorganisms [1]. The balance of the GI microbiota is affected by several factors, but the most important is diet. Polysaccharides obtained from plants, algae, fungi, and bacteria have been established as important modulators of the GI microbiota, particularly in terms of their prebiotic properties [[2], [3], [4], [5], [6]]. Prebiotics are compounds selectively utilized by host microorganisms, thereby providing health benefits, and Polysaccharides have been found to support the growth of beneficial microorganisms such as *Bifidobacteria* and *Lactobacillus* [7].

**Fig. 1:**
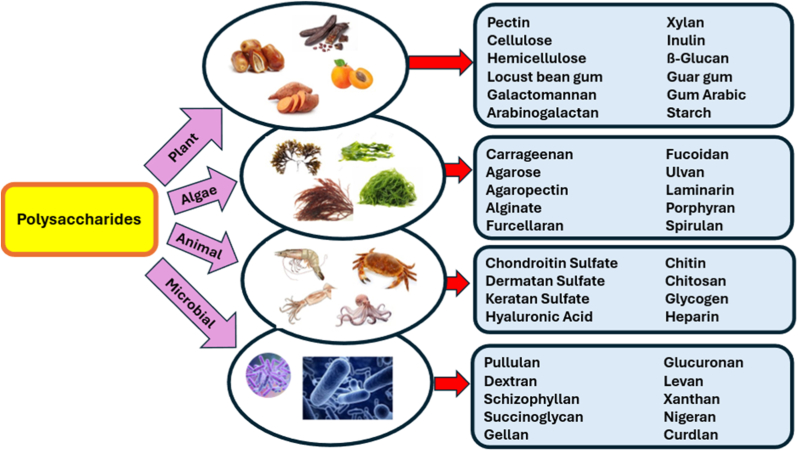
Classification of polysaccharides, according to their biological origin (plant, animal, algal, and microbial) and associated structural and functional diversity.

As structurally diverse biological macromolecules, polysaccharides are composed of monosaccharide units linked by glycosidic bonds. These natural polymers vary in monosaccharide composition, chain length, and branching [8] and exhibit antioxidant, antimicrobial, anti-inflammatory, anticancer, immunomodulatory, anticoagulant, gut microbiota (GM) modulation, intestinal protective, and prebiotic activities [[9], [10], [11], [12]]. Polysaccharides are classified based on criteria such as source, monosaccharide composition (heteropolysaccharides/homopolysaccharides), structure (branched/unbranched), and charge (positive/negative). Homopolysaccharides, composed of a single monomer type, are further divided into storage (e.g., starch, glycogen) and structural (e.g., cellulose, chitin) polysaccharides. Glycosaminoglycans (GAGs) are animal-origin heteropolysaccharides containing different monosaccharides [8]. Based on their origin, Polysaccharides are categorized into plant, animal, algal, and microbial Polysaccharides, as illustrated in Fig. 1. Different sources confer distinct functionalities and biological activities [8].

In contrast to other sources, Polysaccharides are stable in structure because of their intermolecular forces, which are resistant to pH and temperature changes [3,8,13]. Polysaccharides generally have positive effects on gut microbiota, which promotes the growth of beneficial bacteria important in digestion, nutrient production, and immune system modulation [14]. Polysaccharides are not digested and reach the colon, where they stimulate the growth of beneficial bacteria and increase the production of SCFAs, meeting all criteria for prebiotics [15]. Polysaccharides fermentation enhances bowel health, prevents diseases, and eliminates toxins in the body. Prebiotics are described as “non-digestible food ingredients that beneficially affect the host by selectively stimulating the growth and/or activity of one or a limited number of bacteria in the colon, thus improving host health” [16]. The mechanisms by which prebiotics function include changing the composition or function of the intestinal flora, improving digestion, supporting natural defenses, increasing mineral absorption, and regulating appetite [16]. Prebiotics also display antagonistic activity against pathogenic flora by changing the composition of intestinal flora and increasing microbial metabolites such as SCFAs.

The diversity in Polysaccharides, such as the types of monosaccharides, glycosidic linkages, branching, molecular weight, etc., is known to have an impact on the functional properties of Polysaccharides, as well as their interactions with the microbiota, which have been reviewed in detail [[17], [18], [19], [20], [21]]. For instance, β-glucans derived from oats and barley have been shown to promote the growth of SCFA-producing bacteria, while pectin, derived from fruits, is known to stimulate the proliferation of *Akkermansia muciniphila*, a microorganism known for its positive impact on the intestinal barrier function [22].

The role of the gut microbiota in human health is not limited to the digestive system, but includes the metabolic, neurological, and immunological systems." Various diseases, such as obesity, type 2 diabetes, inflammatory bowel disease, and neurodegenerative diseases, have been associated with dysbiosis, or an imbalance in the gut microbiota, [23]. Modulating the gut microbiota with polysaccharides of different origins is believed to have the potential for health benefits and the prevention of diseases. Various research papers have been published concerning the structure and properties of Polysaccharides, as well as their biological activities, in relation to the intestinal barrier [20,21,24]. Other papers have focused on the impact of Polysaccharides of specific dietary origins on the modulation of the gut microbiota, with an emphasis on the health benefits, particularly in relation to in vitro cell cultures and *in vivo* animal models [1,2,6,13,18,19,21,[24], [25], [26], [27]].

Although various reviews have been conducted on the prebiotic properties of dietary Polysaccharides, most of these reviews have concentrated on specific aspects of prebiotic properties, such as microbiological properties and technological properties, individually. A comprehensive framework that considers all these properties and their relationships is not well developed. This review aims to critically evaluate and synthesize the current knowledge on the role of Polysaccharides from various biological sources, including plants, algae, animals, and microbes, in modulating the composition and function of the gut microbiota. This review attempts to highlight the structure-function relationships of Polysaccharides, including their fermentability, selectivity, and bioactivity, based on the findings of various in vitro, *in vivo*, and human intervention studies. It also attempts to highlight the molecular basis of gut microbial modulation and the implications of Polysaccharides in various metabolic and inflammatory diseases, and to discuss the advances and challenges in the extraction and validation of Polysaccharides for their prebiotic properties and applications. This review also attempts to highlight the relationships between the structural features of Polysaccharides and their effects on gut microbiota composition and function, and to discuss the emerging concepts of personalized nutrition and Polysaccharides, and the sustainable production of Polysaccharides and their prebiotic properties and applications.

## 2 Significance of gut microbiota homeostasis and polysaccharide-based interventions

The gut microbiota has a complex composition that varies from person to person. The advantageous bacteria offer certain advantages to the host. For instance, *Bacteroides fragilis* helps in improving the intestinal barrier function and integrity [28]. On the other hand, pathogenic bacteria such as *Clostridium difficile* can cause intestinal mucosal necrosis due to the production of toxins [29]. It should be noted that not all bacteria in the gut can be categorized as advantageous or pathogenic. The activity of gut bacteria depends on the composition and status of the gut. For example, *Akkermansia muciniphila* can improve metabolic diseases but can worsen infections caused by *Salmonella* Typhimurium [30]. Therefore, considering that the increase in pathogenic bacteria and the reduction in advantageous bacteria are common in all diseases, gut microbiota balance restoration is considered a major mechanism through which polysaccharides play a role in ameliorating diseases. Impaired glucose tolerance is strongly correlated with reduced *Lactobacillus* and *Bifidobacterium* bacteria [20], while certain Polysaccharides can increase the number of these bacteria, thereby improving diabetes phenotypes. For instance, *Lactobacillus paracasei* and *Akkermansia muciniphila* can grow better in the presence of polysaccharides from *Gastrodia elata* [31]. Prebiotic regulators have also been shown to increase the population of *A. muciniphila*, Blautia spp., and Alloprevotella spp. in mice, leading to the alleviation of intestinal malnutrition and metabolic syndrome [32]. Polysaccharides from *Crataegus pinnatifida* have been shown to enhance the growth of *Bifidobacterium longum*, Bifidobacterium ovatus, and *Bacteroides thetaiotaomicron* [33]. There is a significant correlation between obesity and the presence of pathogenic bacteria such as *Sutterella*, Desulfovibrionaceae, Streptococcaceae, and *Clostridium* species [34]. *Escherichia coli* is associated with colon cancer and Crohn's disease. However, soluble plantain-derived non-starch Polysaccharides have been shown to inhibit the adhesion of *Escherichia coli* to the mucosa of the intestine, leading to the prevention of inflammatory bowel disease. Non-starch polysaccharides from Musa spp. inhibit the growth of *Salmonella enterica* Typhimurium in the chicken gut [35]. In addition, prebiotics such as fructans, including inulin, have been shown to increase the growth of beneficial bacteria such as *Lactobacillus* and *Bifidobacterium* species [36].

Polysaccharides have a strong regulatory effect on gut microbiota and the immune system; hence, they play a crucial role in the regulation of the microbiota–immune axis. The mechanism through which Polysaccharides exert their activity is mainly based on changes in gut microbiota composition, increased production of microbiota-derived metabolites, and direct and indirect regulation of immune signaling pathways. Polysaccharides supplementation increases SCFAs production, which is a major mediator of immune system regulation and gut homeostasis. These effects are often accompanied by increased levels of SCFA-producing bacteria, including those belonging to the Lachnospiraceae family, *Ruminococcus*, and *Faecalibacterium prausnitzii*, which enhance gut barrier function and anti-inflammatory activity [37,38]. For instance, butyrate induces differentiation of regulatory T cells (Tregs) through histone deacetylase inhibition and activation of G-protein coupled receptors GPR41 and GPR43. These signaling pathways enhance gut barrier function, increasing tight junction protein expression and reducing pro-inflammatory cytokine production, including TNF-α, IL-1β, and IL-6. Propionate and acetate also play a crucial role in immune system regulation through receptor-mediated activity and epigenetic regulation. Through these microbiota-related immune system mechanisms, Polysaccharides exert a synergistic effect on both innate and adaptive immune systems within the microbiota–immune axis [39,40]. Polysaccharides also influence various metabolic pathways, including bile acid metabolism, as well as amino acid and purine metabolism [37,40].

In the case of inflammatory bowel diseases (IBD), a reduction in the number of butyrate-producing bacteria and compromised epithelial layer integrity are common features. Structure-specific polysaccharides that help in the enrichment of butyrate-producing bacteria may prove to be useful as adjunctive nutritional therapies for the treatment of IBD by correcting the microbiota composition and immune homeostasis [41,42]. Nevertheless, the efficacy of polysaccharides-based therapies appears to be subject to significant influence from the composition of the microbiota and disease activity, thus necessitating stratified studies for their assessment.

**Fig. 2:**
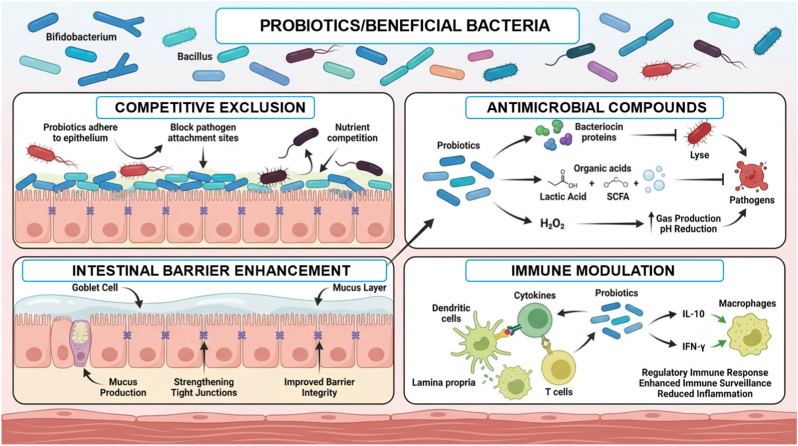
Multifaceted mechanisms of polysaccharides utilized by probiotics in maintaining intestinal homeostasis (The figure was generated with the assistance of https://scispace.com based on author-provided input and subsequently reviewed and edited for scientific accuracy.).

Recently, evidence has suggested that structure-specific polysaccharides may be useful in the treatment of metabolic inflammation and certain autoimmune diseases that involve a state of chronic low-grade inflammation and microbiota dysbiosis [43,44]. However, as with any new therapy, its efficacy in humans remains a challenge due to differences in disease phenotypes, polysaccharides structure, and study designs. A comprehensive understanding of the relationship between polysaccharides, microbiota, and immune system requires the integration of microbiota composition, functional gene expression, metabolomics, and immune biomarkers in the context of a well-designed intervention study. This may help in understanding the role of the structural features of polysaccharides in immune system regulation. Fig. 2 shows the complex mechanisms by which polysaccharides used by probiotics play a role in maintaining gut health.

It is a well-known fact that the equilibrium of gut microbiota is vital for maintaining the health of the host, and polysaccharides-based interventions are considered to be a promising tool for achieving gut microbiota equilibrium by modulating microbiota diversity and density, favoring the production of advantageous metabolites, and boosting immune system function and intestinal barrier function. The efficiency of these interventions is largely dependent on the structural properties of Polysaccharides and their interaction with gut microbiota, which clearly indicates the potential benefits of precision nutrition for the prevention and management of gut microbiota-related diseases.

## 3 Mechanisms of polysaccharide degradation by gut microbiota

**Fig. 3:**
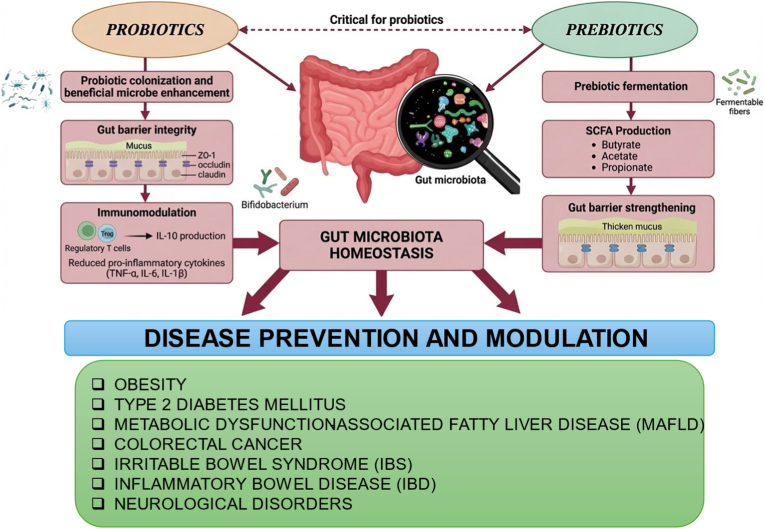
Probiotic-prebiotic interactions in the regulation of gut microbiota homeostasis and disease prevention.

Dietary polysaccharide serve as prebiotics, promoting the diversity, colonization, and persistence of probiotic bacteria, thereby playing a significant role in maintaining gut microbial composition and regulating intestinal homeostasis (Fig. 3) [45,46].

The degradation of dietary polysaccharides by gut microbiota plays a significant role in maintaining host health. The degradation is mediated by a complex interplay between the host's gastrointestinal system and the enzymes produced by the gut microbiota. The degradation is influenced by the polysaccharide's structure and the gut microbiota's composition. Although dietary Polysaccharides are partially broken down by host gastrointestinal enzymes during gastric passage, complex non-starch Polysaccharides are not broken down until they reach the colon [47]. The gut microbiota, particularly those belonging to the phylum Bacteroidetes, are known to produce a wide range of carbohydrate-active enzymes (CAZymes) such as glycoside hydrolases, lyases, and esterase enzymes, which are responsible for degrading complex Polysaccharides into oligo- and monosaccharides [48,49]. The gut microbiota, particularly those belonging to the phylum Bacteroidetes, are known to have specialized genetic loci known as polysaccharide utilization loci (PULs) that are responsible for degrading complex polysaccharides [49].

However, emerging evidence also suggests that pre-colonic gastrointestinal digestion can influence the fermentation of polysaccharides. In some cases, the polysaccharides have been found to undergo partial depolymerization during simulated oral, gastric, and intestinal digestion. This has resulted in the exposure of new sites for microbial fermentation. For example, jujube polysaccharides have been found to undergo partial degradation during simulated gastrointestinal digestion. This has resulted in the enhancement of the production of short-chain fatty acids, the promotion of beneficial microorganisms, and the suppression of detrimental microorganisms during fermentation in the human colon [50]. However, not all polysaccharides have been found to undergo any changes during simulated gastrointestinal digestion. For example, polysaccharides isolated from Zhonghuang No. 1 etiolated green tea have been found to remain intact during simulated gastrointestinal digestion. However, these polysaccharides have been found to be fermented by human fecal microbiota and resulted in the increased relative abundance of Bacteroidota, Faecalibacterium, and Bacteroides, the reduction of the Firmicutes/Bacteroidota ratio, the decrease in pH, and the increase in the production of SCFAs [51]. Digestive transformation of complex preparations has also been found to produce microbiota-active compounds with different effects. For example, digestive transformation products of Changyanning formula have been found to decrease the production of detrimental intestinal gases, increase the production of short-chain fatty acids, decrease Proteobacteria and *Escherichia*/*Shigella*, and increase the relative abundance of beneficial microorganisms such as *Bifidobacterium*, *Lactococcus*, and Faecalibacterium [52].

Microbial degradation proceeds through sequential primary and secondary degradation phases. Primary degraders, such as Bacteroides hydrolyze complex structures into fermentable sugars, and secondary degraders ferment them, producing metabolites important for host health [48,53]. Furthermore, the cross-feeding mechanism is evident in this process, as resource competition and mutualistic interactions among the microbial community promote enhanced diversity and functional complexity [48]. The polysaccharides degradation mechanisms are governed by structural attributes of the Polysaccharides, such as molecular weight, degree of branching, glycosidic bond types and monosaccharide composition. For instance, polysaccharides characterized by lower MW and extensive branching, exhibits rapid fermentation due to enhanced accessibility of microbial enzymes [53,54]. Some polysaccharides, such as sulfated ones, are resistant to degradation by most of the gut microbiota, and only substrate-specific strains selectively degrade them, leading to unique microbial modulations [47]. Overall, the structure and quantity of dietary Polysaccharides can alter the gut microbiota abundance and composition, thereby promoting beneficial strains while suppressing pathogens [49,55].

**Fig. 4:**
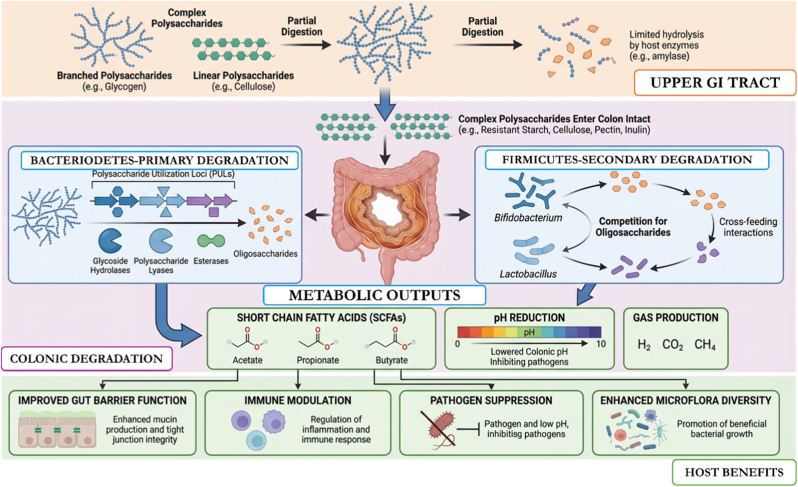
Mechanisms of polysaccharide degradation by gut microbiota and the resulting interactions between microbial enzymes, fermentation products, and host physiological functions (The figure was generated with the assistance of https://scispace.com based on author-provided input and subsequently reviewed and edited for scientific accuracy).

The metabolites, particularly SCFAs (acetic acid, propionic acid, and butyric acid), resulting from polysaccharides degradation and fermentation by gut microbiota are absorbed by the host and contribute to various metabolic pathways, including immune modulation, energy metabolism and gut health. Moreover, the degradation mechanisms generate bioactive metabolites that can influence disease outcomes, interfering with the overall host health [49,53]. Fig. 4 illustrates the sequential degradation of complex polysaccharides by gut microbiota, leading to SCFAs production, reduced colonic pH, and enhanced gut barrier, immune, and microbial homeostasis that collectively confer host health benefits.

## 4 Polysaccharides from diverse sources in gut microbiota modulation: A structure-function perspective

### 4.1 Plant-origin polysaccharides and gut microbiota

Polysaccharides of plant origin can be classified into two groups: storage polysaccharides (starch) and cell wall polysaccharides, also known as non-starch polysaccharides (NSPs). The activity of pancreatic α-amylase enables the hydrolysis of starch in the small intestine, producing glucose that can be absorbed into the bloodstream. NSPs are undigestible in vitro and are fermented in the large intestine by colonic microbiota [56]. Following the advice of the Food and Agriculture Organization of the United Nations and the Codex Alimentarius Commission, plant polysaccharides (both soluble and insoluble) that are not completely hydrolyzed by human digestive enzymes—cellulose, hemicellulose, pectin, gums, and lignified materials—have been classified as dietary fiber [57].

#### 4.1.1 Pectin

**Fig. 5:**
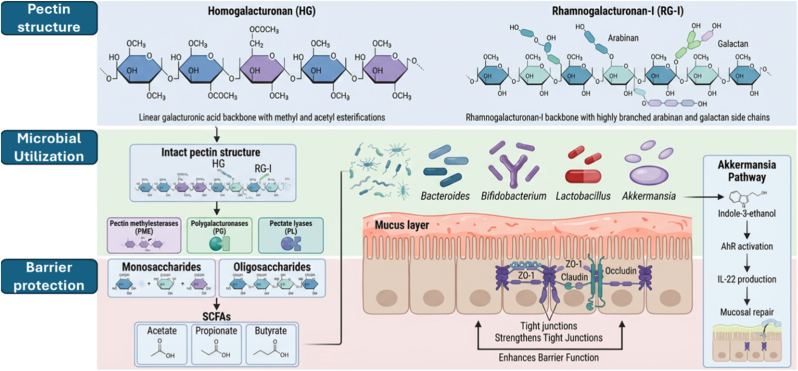
Structure-function relationship of pectin and its influence on gut microbiota modulation, highlighting microbial utilization pathways and barrier-protective effects (The figure was generated with the assistance of https://scispace.com based on author-provided input and subsequently reviewed and edited for scientific accuracy.).

Pectin is one of the main polysaccharides in plant cell walls and is widely used in the food industry as a gelling and thickening agent in foodstuffs like jams and jellies and frozen desserts in which gelation occurs in the presence of Ca2+ ions or in acidic conditions. The bioactivity and prebiotic properties of pectin are greatly affected by its chemical composition, particularly its degree of methylation/esterification (DM/DE), molecular weight (MW), and composition and complexity of its side chains, including its rhamnogalacturonan I (RG-I) and rhamnogalacturonan II (RG-II) regions and neutral sugars arabinose and galactose [58,59]. Its chemical composition consists mainly of a backbone of α-(1 → 4)-linked d-galacturonic acid units and regions of rhamnogalacturonan that have side chains that contain arabinose and galactose units. The composition of pectin varies depending on the source material. Citrus peel pectin has a higher degree of esterification compared to apple pomace pectin, which has more arabinan regions [60]. These structural variations directly affect its physicochemical characteristics, fermentability, and interaction with intestinal microbiota Fig. 5. For instance, low DE pectin (<50%) is more susceptible to hydrolysis due to its high proportion of free carboxyl groups that facilitate better access for microbes, unlike high DE pectin that often requires microbes with esterase activity for effective hydrolysis. Pectin molecular weight is another important factor that significantly influences pectin hydrolysis. For instance, low molecular weight pectin is readily utilized by intestinal microbes such as Bacteroides and Bifidobacterium, whereas high molecular weight pectin is hydrolyzed slowly and is often associated with the growth of Ruminococcus [61,62]. The composition of monosaccharides in the RG domains is another factor that influences pectin hydrolysis selectivity. For instance, pectin rich in arabinan is preferentially utilized by *Bacteroides thetaiotaomicron*, whereas pectin rich in galactan is closely associated with Bifidobacterium growth [63]. Pectin's interaction with intestinal microbiota is closely associated with its detailed structural features.

#### 4.1.2 Cellulose

Cellulose is an insoluble dietary fiber that increases stool bulk and regulates bowel movements. It is a linear homopolysaccharide made of β-(1 → 4)-linked d-glucopyranose units [64]. In plant tissues, cellulose is a structural component of the cell wall that imparts rigidity and strength to the cell wall. The glucan chains aggregate to form microfibrils that associate with other polysaccharides like hemicelluloses and pectin to form a compact structure. The organization of these structures and their association with other cell wall compounds determine the mechanical properties of the cell wall, thereby affecting plant growth, disease resistance, and stress tolerance [65].

Hemicelluloses consist of xylans, mannans, β-glucans, and xyloglucans and have a branched and heterogeneous structure. Hemicelluloses have an amorphous structure and are less ordered compared to cellulose, making them easier to hydrothermally treat and extract from cell walls [66]. The presence of β-1,4-glycosidic bonds between glucose molecules in cellulose contributes to the crystallinity of cellulose and resistance to hydrolysis, thereby affecting water solubility and rendering cellulose insoluble and indigestible by humans. However, certain species of Ruminococcus, Bacteroides, and Firmicutes can hydrolyze cellulose through the production of cellulase enzymes. However, the presence of higher degrees of crystallinity in cellulose makes it difficult for these microbes to ferment cellulose [67,68].

Despite the fact that cellulose is indigestible, it is an essential dietary fiber that regulates and supports digestive health by facilitating regular bowel movements, treating constipation, and reducing the risk of colorectal cancer. Although undigested, cellulose traverses the small intestine and is fermented by colonic microbes to produce SCFAs like acetate, propionate, and butyrate, which serve as energy sources for colonocytes and play a role in luminal pH and immune modulation [69,70].

The accessibility of microbes to cellulose depends on its structure, which involves the formation of microfibrils and interactions with lignin and hemicellulose. This may limit the breakdown of cellulose by enzymes. However, cellulose in complex plant materials may require consortia of microbes that can produce a variety of enzymes to ensure its breakdown. Therefore, the structure of cellulose and its derivatives, such as nanocellulose, determines the diversity of microbiota in the human gut and its fermentation activity [71,72].

**Fig. 6:**
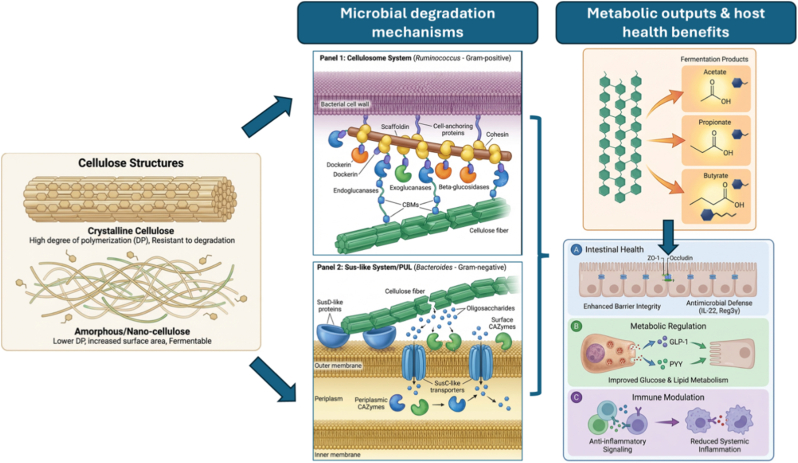
Structure-function relationship of cellulose and its mechanism in gut microbiota modulation (The figure was generated with the assistance of https://scispace.com based on author-provided input and subsequently reviewed and edited for scientific accuracy).

Fig. 6 shows an overview of the structure of cellulose, its breakdown mechanisms by microbes, and its beneficial role in the host. The structure of cellulose, which has a high degree of polymerization in crystalline cellulose, shows higher resistance to enzyme breakdown compared to amorphous or nano-cellulose structures that are easily degraded in the human gut. The breakdown of cellulose in the human gut involves cellulosome complexes in Gram-positive bacteria and Sus-like/PS utilization locus (PUL) systems in Gram-negative bacteria to produce SCFAs. The key cellulolytic bacteria from the Firmicutes phylum and other genera such as Ruminococcus and Bacteroides secrete enzymes such as endocellulases, exocellulases, and β-glucosidases to break down cellulose to produce SCFAs. This shows that the breakdown of cellulose in the human gut depends on the composition of microbiota, which may be influenced by dietary, environmental, and genetic factors; high-fiber diets can stimulate cellulolytic bacteria to produce more SCFAs [73].

Beyond direct cellulose fermentation, the gut microbiota also modulates the host response to cellulose-derived metabolites. This acidification also improves the integrity of the gut. Moreover, the produced butyrate, a member of the SCFAs family, possesses anti-inflammatory properties, creating a balance between pro-inflammatory and anti-inflammatory signals. The diet also modulates the capacity of the microbiota to ferment cellulose. For example, a diet rich in fiber increases the number of cellulolytic microbes, but a diet low in fiber reduces the production of SCFAs [74,75].

To comprehend the interaction between cellulose and the gut microbiome, it is essential to understand the material properties of cellulose. For example, amorphous cellulose is readily degraded, but the presence of crystalline regions makes it difficult for microbial enzymes to break it down, thereby complicating the process of cellulose fermentation [76]. Recent research has also focused on the effect of cellulose structure, solubility, crystallinity, and its integration with the cell wall matrix of the plant cells on the microbiome. Recent research on the microbiome has led to the discovery of genes that contribute to the degradation of cellulose. Elucidating the functional genomics of the microbiome will provide valuable insights into the process of cellulose degradation, thereby improving gut health [67,77].

#### 4.1.3 Resistant starch (RS)

Plant-based foods are known to be a source of starch, which is made up of amylose and amylopectin components. Starch is readily available, inexpensive, and safe for consumption. However, to obtain desirable properties, starch may undergo modification physically, chemically, and/or enzymatically [78]. Starch is one of the most butyrogenic compounds known to man. Epidemiological data show that starchy food consumption is negatively related to the risk of colorectal cancer. The quantity and/or ratio of SCFAs produced during starch fermentation, as revealed by in vitro studies, vary significantly depending on the botanical source of starch. The structural properties are known to affect starch fermentation [79].

Post-modification, several starch properties are enhanced, including mechanical strength, amphiphilicity, hydrophobicity, texture, adhesion, film formation, freeze-thaw stability, thermal stability, and resistance to digestion [80]. Various studies suggest that slowly digestible starch and resistant starch (RS) provide broad health benefits, reducing the risk of diet-related chronic diseases such as diabetes, cardiovascular disease, and obesity [81,82].

Starch acylation has been used to deliver specific SCFAs to the colon consistently and sustainably. Acylated starches, containing RS, serve two functions: (1) direct acylation reduces starch digestibility, and (2) acylation releases additional SCFAs beyond those produced by RS fermentation, as intestinal microbiota liberate the acylated acids [83]. Rat studies have shown that acylated starch effectively delivers significant SCFAs quantities to the colon, offering potential for the treatment and prevention of bowel disorders. Additionally, bacterial groups such as *Ruminococcus bromii*, *Lactobacillus gasseri*, and *Parabacteroides distasonis* may be promoted alongside increases in propionate [84]. The relationship between gut microbiota and type 2 diabetes indices has been explored using native RS from high-amylose maize starch (HAMS) and acylated starch. Acetylated and butylated HAMS enriched *Coprococcus*, *Butyricimonas*, and *Blautia*, suggesting distinct intervention pathways, while propionylated HAMS increased *Bifidobacterium* abundance [85].

Metabolic activity studies comparing HAMS with normal maize starch demonstrated that the highly organized structure of HAMS favoured butyrate production, significantly increasing (P < 0.001) the abundance of key butyrate-producing bacteria, including *Eubacterium hallii* and *Faecalibacterium prausnitzii*. In contrast, normal starch, with a less organized structure, was fermented more quickly, producing more acetate and lactate [79].

Several methods are employed to increase RS content, including enzymatic, chemical, and physical modifications such as etherification, oxidation, and pre-gelatinization [86]. The interaction between RS types and digestive enzymes in the upper gastrointestinal (GI) tract is a key factor in defining RS. Still, it does not fully explain its impact on gut microbiota. Consuming the same RS type can lead to distinct shifts in gut microbiota composition and fermented metabolite profiles [87].

**Fig. 7:**
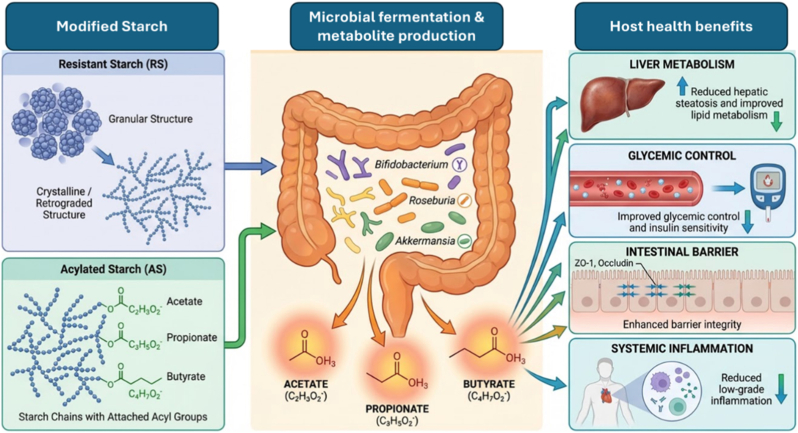
Resistant and acylated starches: structure-driven modulation of gut microbiota and host metabolism (The figure was generated with the assistance of https://scispace.com based on author-provided input and subsequently reviewed and edited for scientific accuracy).

RS is in the limelight for its potential in maintaining the health of the colon, as it reduces the pH, increases the production of SCFAs, particularly acetate and butyrate, while promoting the growth of beneficial microorganisms. Starches modified with propionate and butyric acid have demonstrated the potential of providing SCFAs in the colon in a predictable manner [88]. Polyphenols may also have an impact on starch, such that they can pass through the gastrointestinal tract in an unaltered form. Modification of starch-based carriers through enzymatic or physicochemical means may result in the controlled delivery of polyphenols, thereby protecting the polyphenols in the upper GI tract, affecting the composition of the GI microbiota [86]. Fig. 7 shows the impact of variations in the structure of resistant starch and its esterified forms on the fermentation patterns of the GI microbiota, resulting in the production of SCFAs, thereby improving the metabolism, glycemic status, intestinal integrity, and inflammation in the host.

#### 4.1.4 Plant gums

Plant-based raw materials are used to extract various types of plant gums. These hydrophilic polysaccharides serve as valuable food additives, offering thickening, stabilization, and emulsification properties in food formulations [89]. Arabic gum, a high-molecular-weight heteropolysaccharide, ferments distally in the colon, where many chronic colonic diseases originate, making it a promising prebiotic candidate [90].

Supplementation of Nile tilapia (*Oreochromis niloticus*) with Arabic gum enhanced midgut *Bacillus* abundance and improved antibacterial activity against pathogenic bacteria over a 56-day feeding period [91]. Co-supplementation of Arabic gum and Baobab fiber produced synergistic prebiotic effects, including increased short-chain fatty acids (SCFAs) production, enhanced Akkermansiaceae and Christensenellaceae abundance in the distal colon, and elevated levels of spermidine, serotonin, and ProBetaine, despite a lower dosage strategy (2.5 g/day) [90].

Guar gum, extracted from *Cyamopsis tetragonolobus* seeds, is an indigestible, soluble galactomannan polymer widely used in dairy and bakery products due to its ability to form highly viscous solutions at low concentrations. It is present in ice cream, yoghurt, salad dressing, gluten-free baked goods, sauces, kefir, and breakfast cereals. Its rheological properties help regulate nutrient absorption in the small intestine, benefiting glycemic control and cholesterol metabolism [92]. Partially hydrolyzed guar gum (PHGG), including high- and medium-molecular-weight variants (HHGG and MHGG), improves fecal moisture and small intestinal transit. The highest dose of MHGG resulted in increased production of SCFAs. Similarly, PHGG, together with galactooligosaccharides (GOS) and xylooligosaccharides (XOS), suppressed the abundance of *Desulfovibrio* while promoting the enrichment of Bacteroidetes [93].

Guar gum initially increased anal gas evacuations and digestive sensations, which normalized with continued use, although sensations only partially recovered. Guar gum caused modest changes in microbiota composition, correlating with positive hedonic sensations, including the proliferation of *Agathobaculum butyriciproducens* and *Lachnospira pectinoschiza* [92]. It mainly enriched Actinobacteria, particularly *Bifidobacterium*, and altered genera from Firmicutes and Bacteroidota. In GuD-fed mice, these microbiota changes led to luminal accumulation of lactate and succinate, decreased tight junction markers and colonic IL-18, and increased susceptibility to colitis, although recombinant IL-18 mitigated this effect [94].

Locust bean gum (LBG), derived from the endosperm of carob seeds (*Ceratonia siliqua* L.), is a galactomannan composed of β-1,4-linked d-mannose and α-1,6-linked d-galactose with a mannose-to-galactose ratio of 4:1. LBG functions as a non-digestible soluble fiber and exhibits antioxidant, anti-diabetic, anti-cancer, and anti-obesity properties. Its dietary fiber content aids bowel movement and helps prevent colon cancer [95,96]. Locust bean gum hydrolysate (LBGH), containing monosaccharides and oligosaccharides with degrees of polymerization (DP) from 2 to 7, reduced colonic pathological damage, suppressed pro-inflammatory factor production, increased tight junction protein expression, and promoted the growth of probiotics such as *Lactobacillus* and *Bifidobacterium* [95].

**Fig. 8:**
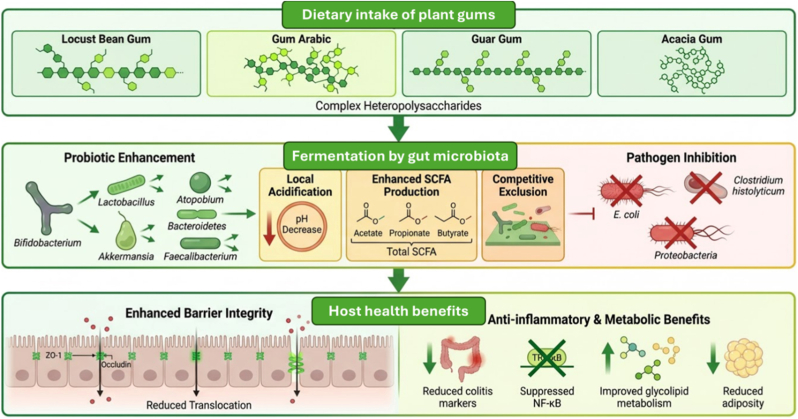
Functional effects of plant gums and their derivatives on gut microbiota modulation, demonstrating enhancement of probiotic populations and inhibition of pathogens (The figure was generated with the assistance of https://scispace.com based on author-provided input and subsequently reviewed and edited for scientific accuracy).

Structural modifications of LBG using Man5HJ14 and ManAJB13 produced manno-oligosaccharides (MOS) ranging from mannose to mannoheptose. These MOS supported the in vitro growth of *Lactobacillus plantarum* CICC 24202, with a preference for smaller MOS (mannose to mannotriose) over larger structures [97]. Further, β-mannooligosaccharides (β-MOS) separated by size exclusion chromatography revealed that DP2 and DP3 oligosaccharides enhanced the growth of three out of seven tested *Lactobacillus* species, whereas DP5 suppressed growth. Additionally, DP2, DP3, and DP5 inhibited *Salmonella typhi*, *Listeria monocytogenes*, and *Escherichia coli* under in vitro conditions [98]. Fig. 8 summarizes the major functional effects of plant gums on gut microbiota modulation.

#### 4.1.5 Inulin

Inulin, a prebiotic naturally found in various plants and vegetables, is currently being studied for its potential to modulate gut microbiota and improve human health. Recent findings show that inulin increases Bifidobacterium levels and enhances metabolic functions, potentially benefiting several metabolic disorders, including obesity, type 2 diabetes, kidney disease, intestinal illnesses, and non-alcoholic fatty liver disease [[99], [100], [101], [102], [103]]. Inulin is classified as a fructan, which consists of linear chains of fructose monomers connected by β-(2 → 1) or β-(1 → 2) glycosidic linkages, typically terminating with a glucose unit at the reducing end [104]. Due to its resistance to digestion in the upper gastrointestinal tract, inulin reaches the colon intact, where it is fermented by the gut microbiota [105].

The degree of polymerization (DP) of inulin varies from 2 to more than 60 fructose units. DP of inulin was considered to be a major determining factor for its fermentability, selectivity, and metabolite production in the colon. Inulin with a low DP is rapidly fermented in the proximal colon, which stimulates saccharolytic bacteria. This produces acetate and lactate early in the process. Inulin with a high DP is fermented more slowly. This sustained fermentation produces SCFAs. Although there is interindividual variation in the composition of the microbiota, functional redundancy among inulin-utilizing bacteria maintains a sustained fermentation pattern and production of SCFAs [104,105].

#### 4.1.6 β-Glucan

Plant-derived β-glucans are mainly found in cereal grain cell walls, including oats (*Avena sativa*), barley (*Hordeum vulgare*), and rye (*Secale cereale* L.). These components play a crucial role in maintaining cell wall integrity; they are linear, mixed-linkage β-(1 → 3),(1 → 4)-D-glucans. Although β-glucans are plant-derived polysaccharides, they are also found in fungi and yeast; these polysaccharides have specific β-(1 → 3)/(1 → 6) configurations compared to cereal β-glucans. The degree of branching and type of linkages influence the physicochemical properties, enzymatic degradation, and fermentability by gut microbiota. For example, algal-derived β-glucans contain mainly β-(1 → 4) linkages; these polysaccharides are less degraded by gut microbiota, while structurally diverse β-glucans from various sources are degraded by a wide range of saccharolytic bacteria [106,107].

It was also reported that oat-derived β-glucans selectively modulate gut microbiota composition by increasing specific genera of β-glucan-degrading bacteria, including *Lactobacillus* and Bacteroides, through specific glycoside hydrolase activity [108]. In addition to oat-derived β-glucans, β-glucans from various sources also increased specific genera of gut microbiota, including Bifidobacterium, Akkermansia, Bacillus, Prevotella, Megamonas, Faecalibacterium, which improved metabolic function [109].

### 4.2 Algal origin polysaccharides and gut microbiota

Marine macroalgae species, such as Porphyra, Ulva, Ascophyllum, Palmaria, and Gracilaria, are good sources of structural (cell wall) polysaccharides (PS), storage polysaccharides, and mucopolysaccharides. The content of polysaccharides in seaweed varies from species to species, ranging from 4% to 76% dry weight. Of particular interest as polysaccharides derived from seaweed for industrial use are alginates, agar, ulvan, laminarin, furcellaran, carrageenan, and fucoidans [110,111]. Apart from taxonomic origins, the response of microbiota to algal polysaccharides is significantly affected by other structural characteristics. For alginate polysaccharides, the ratio of mannuronic to guluronic acids, block distribution, and chain size all influence chain flexibility, enzymatic accessibility, and rates of fermentation; thus, alginate oligosaccharides generally exhibit faster rates of utilization than high-molecular-weight alginate polymers. In carrageenans, polysaccharide sulfation levels and polymerization levels significantly influence response to polysaccharide fermentation; in fact, oligosaccharide levels of κ-carrageenan polysaccharides containing low levels of polymerization might exhibit greater beneficial effects than those polysaccharides that are highly sulfated and less readily fermented. Fucoidan polysaccharide functionality is also subject to various factors such as linkage type, levels of sulfation, as well as levels of polysaccharide chain weight; thus, it is important to view algal polysaccharides as structure-defined rather than as a single category of polysaccharide sources.

Algal-derived polysaccharides, such as fucoidan, carrageenan, and alginates, play a critical role in modifying gut microbiota composition. Since human digestive enzymes cannot degrade these polysaccharides, they arrive at the large intestine as a whole, acting as a prebiotic. It has been demonstrated that algal-derived polysaccharides can increase the number of beneficial bacteria (such as *Bifidobacteria* and *Lactobacillus*) while reducing the number of potentially pathogenic bacteria (such as *Clostridium* and Enterobacteriaceae). This gut microbiota shift increases the production of SCFAs such as butyrate, propionate, and acetate, which play a critical role in maintaining gut health and inflammation. For example, fucoidan from Brown Algae increases gut microbiota.

Sodium alginate (SA), a water-soluble dietary fiber extracted from brown algae, is a linear polysaccharides that has been employed as a drug delivery system, food stabilizer, and biodegradable film component [112]. SA resists the invasion of enterobacteria in the small intestine and can be efficiently fermented by intestinal bacteria, especially increasing Bacteroides bacteria and SCFAs in the colon [113]. SA supplementation improved intestinal barrier function, immune responses, and gut microbiota composition in cyclophosphamide-induced immunosuppressed mice models by reducing the proportion of detrimental bacteria such as *Helicobacter*, Peptococcus, Tyzzerella, while increasing the proportion of beneficial bacteria such as *Lactobacillus*, Roseburia [114].

Alginate oligosaccharides (AOS), including guluranate (GAOS), mannuronic (MAOS), and heterozygous alginate oligosaccharides (HAOS), were found to possess ameliorative properties in DSS-induced mice models with colitis by maintaining colon length, reducing disease activity, increasing SCFAs, and modulating gut microbiota composition [115]. Among them, MAOS showed better efficacy in alleviating DSS-induced colitis symptoms and improving gut barrier function in vitro and *in vivo*, indicating its potential for the treatment of gut-related diseases [116]. In addition, alginate was found to partially improve gut microbiota richness and diversity after ovalbumin-induced gut microbiota disruption, with increased relative abundance of beneficial bacteria including Alloprevotella, Bacteroides, Parabacteroides, Rikenellaceae_RC9_gut_group [117].

Carrageenans are linear sulfated polygalactans of red algae (Rhodophyceae) that are classified based on the sulfation pattern as κ (kappa), ι (iota), and λ (lambda) carrageenans [118]. They are commonly used as food stabilizers and gelling agents but are also being investigated for potential use in drug delivery systems. Carrageenan ingestion in mice models has been found to alter the microbiota composition by increasing Bacteroidota without significant changes in the microbiota composition but affecting carbohydrate metabolism and increasing fecal cytotoxicity due to oxidative stress and immunosuppression [119]. High λ-carrageenan ingestion has also been found to decrease the colonic mucus layer integrity, increase fecal LPS levels, and decrease SCFAs produced, which can be transferred to other mice models through FMT [120]. However, another study found that ingestion of λ-carrageenan improved memory function and altered the microbiota composition to decrease the production of harmful compounds and increase butyric acid production regardless of its molecular weight [121]. The κ-carrageenan oligosaccharides KO3 and KO6 were found to have different in vitro fermentation profiles; KO3 was found to support the growth of *Lactobacillus* and Bifidobacterium and increase SCFAs produced, whereas KO6 was found to decrease SCFAs produced and increase Prevotellaceae levels, which are linked to mucin degradation [122].

Fucoidan, primarily composed of l-fucose, is extracted from brown algae cell walls. It has demonstrated benefits including lipid metabolism regulation, anticoagulant effects, gastric protection, and anti-inflammatory activities. The bioactivity of fucoidan is heavily influenced by its structure, which varies with algal species. The fucoidan from *Fucus vesiculosus*, with a relatively simple backbone of α-(1 → 3)- and α-(1 → 4)-linked fucose residues, is the most extensively studied [123].

In mice with dextran sulfate sodium (DSS)-induced colitis, the impact of fucoidan on gut microbiota and bile acid metabolism was examined. Fucoidan successfully restored the gut barrier, reduced colonic inflammation, and increased SCFAs production, particularly butyrate. It also elevated the abundance of the Lachnospiraceae family, including *Turicibacter*, *Muribaculum*, *Parasutterella*, and *Colidextribacter* [124]. In another study, *Undaria pinnatifida* fucoidan improved dyslipidaemia and altered gut microbiota in BALB/c mice fed a high-fat diet. Fucoidan increased Bacteroidetes and *Alloprevotella* abundance while reducing Firmicutes, *Staphylococcus*, and *Streptococcus* [125].

Fucoidan's therapeutic potential in inflammatory bowel disease (IBD) was further demonstrated in DSS-induced models. Purified fucoidan, containing →3)-β-D-Galp-(1→, →4)-α-L-Fucp-(1→, and →3)-α-L-Fucp-(1→) residues with C2 sulfation, reduced IBD symptoms, restored gut microbiota diversity by increasing Bacteroidetes and reducing Firmicutes, and decreased levels of pro-inflammatory cytokines such as IL-1β, IL-6, TNF-α, and IFN-γ [126]. *Acaudina molpadioides* fucoidan (Am-FUC) altered gut microbiota by reducing Firmicutes and increasing Bacteroidetes, significantly improving insulin resistance. Am-FUC decreased serum and fecal lipopolysaccharide (LPS) levels, inhibited toll-like receptor 4 signalling, activated adenosine monophosphate-activated protein kinase, and likely elevated G-protein-coupled receptor 43 levels, correlated with increased fecal SCFAs [127].

The marine green macroalga *Ulva ohnoi* is rich in ulvan, a sulfated Polysaccharides composed of sugars, such as xylose, rhamnose, and various uronic acids. Ulvan has been explored for its nutraceutical, antioxidant, anti-inflammatory, and immunomodulatory properties [128].

Ulvan acts as a prebiotic by serving as a fermentable substrate for beneficial bacteria such as *Bifidobacterium* and *Lactobacillus*. These bacteria ferment ulvan to produce SCFAs (acetate, propionate, and butyrate), which lower gut pH, inhibit pathogens like *Escherichia coli* and *Clostridium perfringens*, strengthen intestinal barrier function, and reduce inflammation [26,129]. Ulvan's sulfated structure also contributes to its immunomodulatory effects by promoting anti-inflammatory cytokines (e.g., IL-10) and suppressing pro-inflammatory cytokines (e.g., TNF-α, IL-6). This creates a favorable environment for beneficial bacteria while directly inhibiting pathogenic bacteria through membrane disruption and adhesion inhibition [130]. Combined supplementation with ulvan and astaxanthin from *Ulva ohnoi* and *Haematococcus pluvialis* altered murine gut microbiota, increasing the abundance of *Bacteroidia*, *Bacilli*, *Clostridium*, and Verrucomicrobia [131].

*Ulva lactuca* polysaccharide (ULP) was found to reduce hepatocellular carcinoma tumor growth by altering gut microbial communities (*Tenericutes*, *Agathobacter*, *Ruminiclostridium*, *Parabacteroides*, *Lactobacillus*, and *Holdemania*) and metabolites (e.g., docosahexaenoic acid, uric acid, N-oleoyl dopamine, and l-kynurenine). ULP inhibited key signalling pathways (JNK, c-JUN, PI3K, Akt, and Bcl-6), increased reactive oxygen species (ROS) production, and slowed HepG2 cell growth [132].

### 4.3 Animal origin polysaccharides and gut microbiota

Animal-based Polysaccharides have emerged as intriguing bioactive compounds with significant potential to modulate gut microbiota and promote human health. Unlike plant-derived Polysaccharidess, animal-origin Polysaccharides, such as chitosan, chondroitin sulfate, and hyaluronic acid, possess distinct structural and functional characteristics that uniquely influence gut ecology. These Polysaccharides are cost-effective and sustainable, often sourced as byproducts from the cattle and fisheries industries. Their resistance to digestion in the upper gastrointestinal (GI) tract allows them to reach the colon, where they interact with gut microbiota, inhibiting harmful species and promoting the growth of beneficial bacteria [133].

Chitin, chitosan, and glycosaminoglycans, including dermatan sulfate, hyaluronic acid, keratan sulfate, chondroitin sulfate, heparin, and heparan sulfate, are considered major animal-origin Polysaccharides. These compounds can be extracted from various animal tissues and by-products of the meat and seafood industries [134]. As is the case with algae polysaccharides, it is the structure rather than the source of animal polysaccharides that determines their microbiota-related properties. For chitin and chitosan, it is factors such as acetylation/deacetylation levels, molecular weight, and hence solubility that will determine if these polysaccharides act as slowly fermented material, an easily fermented oligosaccharide, or as a cationic polymer with antimicrobial activity. Low-molecular-weight chitosan derivatives will likely display greater levels of dispersability as well as greater accessibility to microbial enzymes, whereas highly deacetylated chitosan might display greater levels of pathogen inhibition. For glycosaminoglycans, it is factors such as chain length as well as levels of sulfation that will determine enzyme accessibility as well as selectivity of microbes. Thus, while hyaluronic acid is not sulfated, other GAGs such as chondroitin sulfate or heparin, due to their levels of sulfation as well as their varying chain lengths, do not act as equivalent material in terms of microbiota-related properties. As is the case with algae polysaccharides, it is the structure rather than the source of animal polysaccharides that determines their microbiota-related properties. For chitin and chitosan, it is factors such as acetylation/deacetylation levels, molecular weight, and hence solubility that will determine if these polysaccharides act as slowly fermented material, an easily fermented oligosaccharide, or as a cationic polymer with antimicrobial activity. Low-molecular-weight chitosan derivatives will likely display greater levels of dispersability as well as greater accessibility to microbial enzymes, whereas highly deacetylated chitosan might display greater levels of pathogen inhibition. For glycosaminoglycans, it is factors such as chain length as well as levels of sulfation that will determine enzyme accessibility as well as selectivity of microbes. Thus, while hyaluronic acid is not sulfated, other GAGs such as chondroitin sulfate or heparin, due to their levels of sulfation as well as their varying chain lengths, do not act as equivalent material in terms of microbiota-related properties.

#### 4.3.1 Chitin and chitosan

Chitin is an abundantly available structural polysaccharide, which is also present in the exoskeletons of arthropods, as well as in the cell walls of fungi and yeasts. The commercial source of chitin is by-products of seafood industries, including exoskeletons of shrimp, crab, and fish scales. The most prominent derivative of chitin is chitosan, which is synthesized by partial N-deacetylation of chitin under alkaline conditions [135,136]. Chitin and its derivatives are non-toxic, biodegradable, and biocompatible, and there are no environmental hazards involved in their use. The applications of chitin and its derivatives are varied, including food packaging, biomedicine, clothing, cosmetics, and photography.

The potential application of chitin in regulating gut microbiota is also being explored, and research findings show that chitin and its derivatives, including chitosan, act as a prebiotic, promoting the growth of beneficial bacteria such as *Lactobacillus* and Bifidobacterium, which ferment chitin to SCFAs. SCFAs are essential for maintaining gut health, as they increase the strength of the intestinal barrier, reduce inflammation, and decrease pH, making it less favorable for pathogenic bacteria to thrive [137].

In addition to its prebiotic properties, the immunomodulatory and antibacterial properties of chitin have been demonstrated, providing further support for the regulation of the gut microbiota. Specifically, the immunomodulatory properties of chitin have been shown to modulate the innate immune response through its interaction with pattern recognition receptors, such as Toll-like receptors (TLRs) and NOD-like receptors (NLRs), found in immune and epithelial cells in the gut, thereby inhibiting pro-inflammatory cytokines such as TNF-α and IL-6, while stimulating anti-inflammatory cytokine production, such as IL-10. The antibacterial properties of chitin involve the disruption of the cell membranes of bacteria, as well as the inhibition of the adhesion of pathogens to the intestinal epithelium, thereby restricting the proliferation of pathogenic bacteria such as Salmonella spp. and *Escherichia coli* [138].

A further consequence of chitin fermentation is the reinforcement of intestinal barrier integrity, as its supplementation increases the expression of tight junction proteins such as occludin and zonula occludens-1 (ZO-1), thereby strengthening the intestinal barrier, thereby preventing the translocation of toxins and pathogenic microorganisms into the bloodstream. Moreover, the SCFAs, particularly butyrate, generated in the fermentation of chitin, provides energy for the maintenance of the integrity of the intestinal barrier function [137].

#### 4.3.2 Glycosaminoglycans (GAGs)

GAGs are linear, negatively charged, and heteropolysaccharides of animal origin, composed of disaccharide repeating units and having a molecular weight of 10-100 kDa. Hyaluronic acid is a non-sulfated GAG, whereas keratan sulfate, dermatan sulfate, chondroitin sulfate, heparin, and heparan sulfate are sulfated GAGs. GAGs have shown significant potential in treating various diseases, including cancer, pancreatitis, rheumatoid arthritis, nervous system damage, respiratory diseases, and various biotechnological applications [139].

GAGs were previously extracted from terrestrial sources such as swine and bovine byproducts; however, due to bovine spongiform encephalopathy and other diseases, researchers are now looking towards marine sources to obtain these compounds. Marine sources include sponges, sea cucumbers, squids, mollusks, and fish cartilages, and they show various bioactivities such as anticoagulation, immunomodulation, and anti-obesity effects [136,140]. The bioactivity of these compounds is affected by various factors, including molecular weight and structure [135].

GAGs cannot be chemically synthesized and can only be extracted from animals. There is a difference in the molecular weight and sulfation pattern of these compounds when they are extracted from terrestrial and marine sources, affecting their bioactivity and therapeutic potential. This difference in structure influences the interaction of these compounds with proteins, leading to species-specific differences in disaccharide composition, chain length, and sulfation [141].

### 4.4 Microbial origin polysaccharides

**Fig. 9:**
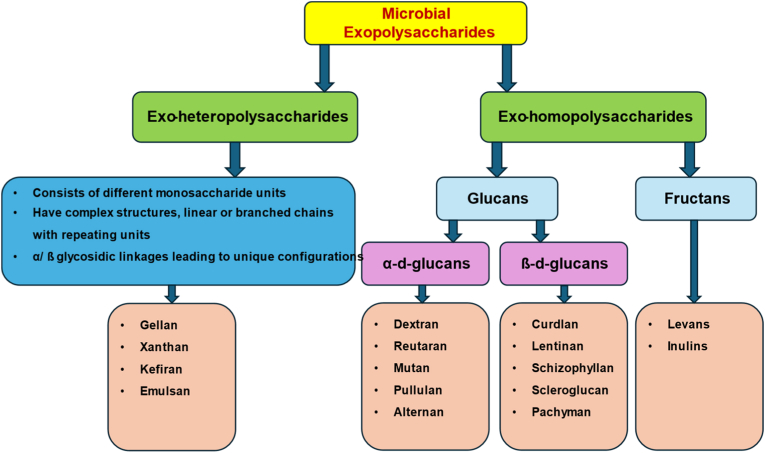
Classification of microbial exopolysaccharides based on structural composition and representative types.

In comparison to plant gums and synthetic Polysaccharides, microbial Polysaccharides have several benefits, such as production regardless of location and climate, ease of extraction, rapid growth rates of microbes, genetic modification of Polysaccharides to attain specific properties, and the utilization of food waste from industries as a cheap and environmentally friendly growth medium [142]. Bacterial Polysaccharides can be broadly categorized into three types: intracellular Polysaccharides, related to cytoplasmic membrane and granules; cell wall polysaccharides; and extracellular polysaccharides, which can be loose slime and tightly bound capsule types [143,144]. Fig. 9 shows the structural composition of microbial exopolysaccharides.

Extracellular polysaccharides are long-chain polymers secreted by bacteria and microalgae. These Polysaccharides are widely used in the food industry as water-binding agents, gelling agents, stabilizers, and emulsifiers. In addition to their technological applications, extracellular polysaccharides are associated with several health-promoting effects, including cholesterol reduction, inhibition of pathogenic biofilm formation, modulation of epithelial cell adhesion and gut microbiota, and antioxidant and anticancer activities.

Extracellular polysaccharides are classified as homopolysaccharides, such as α-D-glucans, β-D-glucans, and β-D-fructans, or as heteropolysaccharides, including kefiran, gellan gum, and xanthan gum, depending on their monosaccharide composition [142,145]. The biological activities of Polysaccharides are strongly influenced by their structural characteristics. Factors such as molecular weight, degree of branching, glycosidic linkages, spatial conformation, and chemical modifications determine their antioxidant, antitumor, immunomodulatory, hypoglycemic, antibacterial, and gut microbiota-modulating properties [146] (Fig. 8).

Xanthan gum is a complex polysaccharides known for its distinctive rheological behavior. It is commonly used as a thickener and stabilizer in processed food products. The chemical structure of xanthan gum differs from that of typical dietary and host Polysaccharides [147]. The safety of low-molecular-weight xanthan gum (LW-XG) has been demonstrated in Caco-2 cells and in mice. Supplementation with LW-XG increased SCFAs concentrations, including acetate, propionate, and butyrate, and enhanced gut microbiota diversity. Notably, the Firmicutes to Bacteroidetes ratio increased from 0.3547 to 1.5371 following supplementation [148]. Xanthan gum is primarily degraded by specialized gut bacteria such as Ruminococcaceae, while *Bacteroides intestinalis* utilizes the resulting oligosaccharides. Studies in germfree mice indicate that xanthan gum supports a cooperative microbial food chain, promoting the growth of key degraders and providing insight into the effects of emerging food additives on human gut microbiota [147].

Dextran is another microbial exopolysaccharide produced by lactic acid bacteria (LAB) or by their enzymes in the presence of sucrose. Dextran is composed of α-(1 → 6)-linked d-glucose units, with occasional branches formed through α-(1 → 4), α-(1 → 3), or α-(1 → 2) linkages. Its molecular weight varies depending on the producing strain [149]. *In vitro* studies demonstrated that dextran and oligodextrans enriched *Bifidobacterium* populations, generated high butyrate concentrations (5–14.85 mmol/L), and supported bacterial growth for up to 48 h. Oligodextran IV, in particular, maintained higher steady-state populations of *Bifidobacterium* and *Lactobacillus* in a continuous gut model compared with dextran and maltodextrin [150].

Gellan gum (GG) is synthesized by *Sphingomonas elodea* and is classified as an anionic polysaccharides. It consists of a linear repeating tetrasaccharide unit composed of β-glucopyranose, β-glucuronopyranose, β-glucopyranose, and α-rhamnopyranose. GG is categorized as either high-acyl or low-acyl according to its acetyl group content, and this characteristic influences its gelation behavior and colloidal stability [151,152]. *In vitro* fermentation studies showed that GG promoted the growth of probiotic strains such as *Bifidobacterium bifidum* and *Lactiplantibacillus rhamnosus*, leading to enhanced SCFAs production. In high-fat diet-fed mice, GG supplementation reduced body fat accumulation, weight gain, blood biomarkers, and hepatic triglyceride levels. It also modulated the gut microbiota, particularly bacterial orders such as *Desulfovibrionales*, *Deferribacterales*, *Bacteroidales*, and *Lactobacillales* [153].

The effects of partially hydrolyzed guar gum (PHGG) with high molecular weight (HHGG, Mw 10,000–30,000 Da) and medium molecular weight (MHGG, Mw 2000–10,000 Da) on gut microbiota regulation and constipation relief were evaluated in a mouse model. After 15 days of intragastric administration, constipation was induced on day 16 using loperamide lavage. PHGG shortened the time to first black stool defecation, increased fecal moisture content, and improved small intestinal transit. The group receiving the highest dose of MHGG produced the greatest amount of SCFAs. Additionally, PHGG, together with galactooligosaccharides (GOS) and xylooligosaccharides (XOS), promoted the accumulation of Bacteroidetes while inhibiting the growth of *Desulfovibrio* [93].

A 56-day feeding trial was conducted to assess the effects of various viscous guar gums on growth, intestinal flora, and intestinal health in *Micropterus salmoides* (largemouth bass). High-viscosity guar gum reduced the digesta butyrate/histamine ratio, increased the abundance of *Plesiomonas shigelloides*, and downregulated the expression of intestinal tight junction, anti-inflammatory, and anti-apoptotic genes. These findings suggest that the viscosity of dietary guar gum should be considered when used as a binder in aquafeeds, as it may negatively affect intestinal health by altering the structure and metabolite profile of intestinal flora [154]. Table 1 summarizes the major polysaccharides sources, structures, and their specific impacts on gut microbial populations.

**Table 1:** Summary of major polysaccharide sources, structures, and their specific impact on gut microbial populations.

Origin	Source/type of polysaccharides	Mw	Structures	Gut benefits (microbe names)	Reference
Plant	PS from mango seed kernels	4.2 × 10^4^ Da	Xylose, fucose, rhamnose, mannose, ribose, glucose, arabinose and galactose	↑ The ratio of Firmicutes to Bacteroidetes ↑ *Prevotella* and *Bacteroides*	[155]
Plant	PS from tea	617.9 kDa	Ribose, galactose, glucose, galacturonic acid, arabinose, xylose, rhamnose, and mannose	↓ Pathogenic bacteria such as *Desulfovibrio* and *Escherichia*, ↑Beneficial genera like *Lactobacillus*, *Bifidobacterium*, and *Prevotella*	[156]
Plant	PS from Fuzhuan brick tea	NA	Galacturonic acid, rhamnose, galactose, arabinose and mannose	↑*Bacteroides ovatus*, *B. uniformis*, *B. fragilis* and *B. thetaiotaomicron*. Transcriptome analysis of *B. ovatus* revealed that 602 genes were up-regulated by FBTPS-3	[157]
Plant	PS from *Auricularia auricula*	1.67 × 10^3^ kDa	Mannose, glucose and xylose	*↑Odoribacter*, *Lactobacillus*, *Dorea* and *Bifidobacterium*. ↓The Firmicutes to Bacteroidetes ratio (F/B)	[158]
Fungal	Two hydrolysates of chitin from inter cellular *Aspergillus cristatus*	985.46 Da	Hydrolats of COS1 and COS2 are mainly composed of oligosaccharides, with the purity of 91.8% and 90.2%	↑the abundance of key bacteria such as *Dubosiella*, *Romboutsia*, and *Turicibacter*. ↓The abundance of key bacteria such as, *Escherichia-Shigella* and *Helicobacter*	[159]
Plant	Tea polysaccharides from Taiping Houkui	782 kDa	GalA, Gal, Ara with little molar contents of Man, Rib, Rha and Glc in the molar ratio of 42.6:20.6:19.4:3.5:2.6:4.4:6.9	↓The ratio of Firmicutes*/*Bacteroidetes, ↑ The growth of *Bacteroides*, and improving the pathway of amino acid metabolism.	[160]
Brown algae	Fucoidan from *Ascophyllum nodosum*	1330 kDa	Man: GluA: Glc: Gal: Xyl: Fuc = 7.3: 24.1: 1.5: 7.2: 1.3: 58.6	↑Verrucomicrobia*, Desulfovibrio, Clostridiales*, and *Bacteroides* *↓*Bacteroidetes and *Rikenellaceae*	[161]
Red algae	Galactan from *Coralline pilulifera*	716 kDa	Gal >90%	↑*Bacteroides, Megamonas,* and *Veillonella*	[162]
Red algae	PS from *Gracilaria lemaneiformis*	22.38 kDa	Ara: Fuc: Xyl: Man: Glc: Gal = 1.14: 1.45: 7.92: 4.23: 13.48: 71.78	↑Firmicutes and Bacteroidetes ↓Proteobacteria, *Fusobacteria*, and *Synergistetes*	[163]
Green algae	Dietary fiber from *Enteromorpha compressa*	NA	Fuc: Glc = 1: 5.94	↑*Lactobacillus* and *Bifidobacterium* ↓*Staphylococcus aureu*, *Shigella flexneri, Escherichia coli,* and *Salmonella typhimurium*	[164]
Seawood	Sodium alginate obtained from Qingdao Bright Moon Seaweed Group	3350 kDa; and 131 kDa	NA	↑ *Faecalibacterium sp. UBA 1819*, ↑*Candidatus_Soleaferrea,* ↑*Faecalitalea,* ↑*Ruminococcus_torques*, ↑*Muribaculum*, ↑*Flavonifractor,* ↑*Parvibacter,* and ↑*Faecelibaculum. Faecalibacterium sp*.	[165]
Animal	Chitin derivatives from the exoskeleton of arthropods	NA	Chitosan (CS), chitooligosaccharides (COS), and glucosamine (GlcN),	↑ *Lactobacillus* and *Bifidobacterium*	[137]
Animal	Mast cells	NA	Linear polycationic glycosaminoglycan (GAG) macromolecule composed of alduronic acid and hexosamine	↑the relative abundance of *Bacteroides* and *Bifidobacterium* and ↓ the relative abundance of *Escherichia*-*Shigella*	[166]
Bacterial	Exopolysaccharide (EPS) from *Lactobacillus rhamnosus*	NA	Four types of EPS with different molecular compositions	↑The abundance of *Ruminococcus, Dorea, Butyricicoccus, and Blautia.* ↓the proportions of *Streptococcus* and *Lactobacillus* *↑*the proportions of *Bacteroides, Escherichia-Shigella and Enterococcus*	[167]
Bacterial	Exopolysaccharides from *Lactobacillus buchneri TCP016*	8.509 × 10^4^ Da	Rhamnose, xylose, glucosamine, glucuronic acid, galactose, galacturonic acid, glucose, and mannose in molar ratios of 9.2:3.9:3.8:2.8:2.1:2.0:1.6:1.0	↓ The enrichment of *Helicobacter*aceae*,* Lachnospiraceae*, and* Enterobacteriaceae ↑, the abundance of *Lactobacillus,* Rikenellaceae*,* Bacteroidaceae*, Bacteroidales_S24-*7_group, and Prevotellaceae	[168]
Bacterial	Exopolysaccharide from *Lactobacillus rhamnosus ZFM231*	1.32 × 10^4^ Da	Mannose, galactose, glucosamine, glucose, glucuronic acid, and rhamnose with molar ratios of 7.08, 2.61, 2.44, 2.22, 1.59, and 1, respectively	↓ Firmicutes*,* ↑Bacteroidetes *and* Actinobacteria *↑*The ratio of the Firmicutes *to* Bacteroidetes *(F/B ratio).* ↑the abundance of *norank_f_Muribaculaceeae, Dubosiella, Bifidobacterium, and* Lachnospiraceae*_NK4A136*_group. ↓the abundance of *Lactobacillus, Streptococcus and Escherichia-Shigella*	[169]

↓-Decrease, ↑-Increase, kDa-Kilo Dalton, Da-Dalton.

Although Polysaccharides derived from plants, algae, animals, and microorganisms differ in their origin and structural complexity, their biological effects tend to converge through common microbial metabolic pathways. Differences observed in dominant bacterial taxa and SCFAs production profiles are mainly attributed to variations in structural accessibility rather than to the source itself. Understanding this shared functional framework supports a more systematic classification of Polysaccharides based on their physiological outcomes instead of solely their botanical or biological origin. This perspective facilitates the translation of mechanistic insights into rational and targeted food design strategies.

### 4.5 Emerging and engineered polysaccharides and gut microbiota

Recent developments in polysaccharides research move beyond traditional dietary supplementation toward engineered and precision-oriented approaches. Engineered Polysaccharides and nanoformulations are being designed to enhance targeted colonic delivery, improve mucoadhesion, and enable stimuli-responsive release systems, particularly in the context of inflammatory bowel disease (IBD) management. At the same time, algal-derived Polysaccharides, including fucoidan, laminarin, and alginate, as well as their structurally modified derivatives, are being recognized as next-generation prebiotics. These compounds exhibit distinct regulatory effects on gut microbiota composition and metabolic activity [170].

**Fig. 10:**
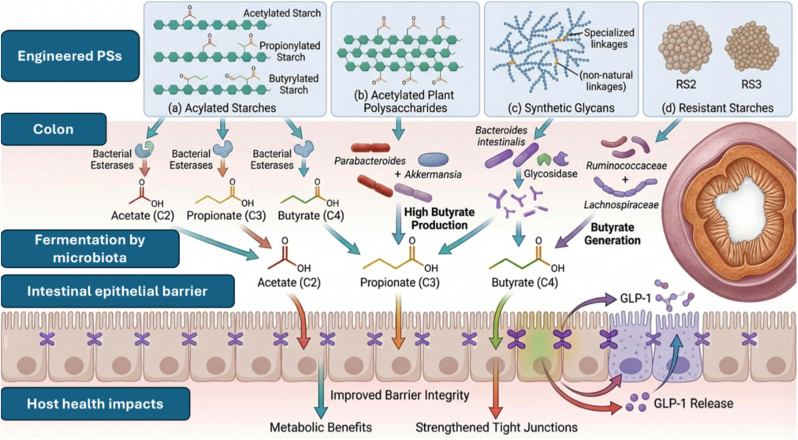
Engineered polysaccharides for targeted microbial fermentation and host metabolic regulation (The figure was generated with the assistance of https://scispace.com based on author-provided input and subsequently reviewed and edited for scientific accuracy).

Despite their promising biofunctional potential, several critical aspects require further evaluation. Issues related to safety assessment, bioavailability, dose optimization, and regulatory approval remain to be systematically addressed. The emerging concept of precision prebiotics aims to customize polysaccharides interventions according to individual gut microbiota profiles in order to maximize therapeutic efficacy. However, important challenges persist, particularly with respect to inter-individual variability in response and the establishment of clear dose–response relationships. These limitations underscore the need for more stratified and mechanistically informed clinical investigations [24,171]. Fig. 10 illustrates the microbial fermentation mechanisms of engineered Polysaccharides, such as acetylated starches, acetylated plant Polysaccharides, synthetic glycans, resistant starches, citric acid-modified starch, and enzymatically modified starch and host health benefits.

## 5 Comparative effects of polysaccharides on gut microbiota

The human gut microbiota is a highly complex microbial community dominated by four major phyla: Gram-negative Bacteroidetes, generalists in glycan catabolism; Gram-positive Firmicutes, specialists in breaking down specific glycans; Proteobacteria; and Actinobacteria, which encode various functions including carbohydrate metabolism and the synthesis of vitamins, amino acids, and cofactors [172]. Carbohydrate-active enzymes (CAZymes) produced by these bacteria degrade dietary Polysaccharides. The colonic microbiota contains a comprehensive array of CAZyme genes, including 136 glycoside hydrolase (GH) genes and 29 polysaccharide lyase (PL) genes [173].

*Bifidobacterium* spp. and *Lactobacillus* spp. are recognized as beneficial microbes producing antimicrobial metabolites that inhibit pathogens and promote gut health [174,175]. Their benefits include boosting immune function, improving digestion and nutrient absorption, synthesizing vitamins, preventing pathogen growth, lowering cholesterol, alleviating gas distension, and restoring gut flora after antibiotic use. SCFAs, major byproducts of polysaccharide metabolism by gut microbiota, have been strongly correlated with increased beneficial bacterial populations in vitro fecal fermentation studies [176,177]. Table 2 presents an overview of SCFAs production profiles induced by Polysaccharides from different origins.

**Table 2:** Comparative analysis of SCFAs production profiles induced by different origin polysaccharides.

Origin	Polysaccharide Source (name)	Assessment Technique	Assessment Model	Analytical Techniques	SCFAs Changes	References
Algae	*Ascophyllum* *Nodosum* (AnPs)	Human fecal fermentation	*In vitro*	GC-FID	↑n-Butyric acid ↑Acetic acid ↑Propionic acid	[161]
Plant	*Lycium barbarum* (LBPS)	Human fecal fermentation	*In vitro*	GC-FID	↑Acetic acid ↑Propionic acid ↑*n*-Butyric acid ↑Lactic acid ↑i-Butyric acid ↑*n*-Valeric acid ↑i-Valeric acid	[178]
Plant	*Porphyra haitanensis* (PHP)	Human fecal fermentation	*In vitro*	GC-FID	↑Acetic acid ↑Propionic acid ↑Butyric acid ↑i-Butyric acid ↑Valeric acid ↑i-Valeric acid ↑Total SCFAs	[179]
Algae	*Coralline pilulifera* (CPPS-3)	Human fecal fermentation	*In vitro*	GC-FID	↑Acetic acid ↑Propionic acid ↓*n*-Butyric acid ↓Lactic acid ↓i-Butyric acid ↓*n*-Valeric acid ↓i-Valeric acid	[176]
Plant	Bamboo shoots (U-CPS; E-CPS)	MRS basal medium fermentation by probiotics	*In vitro*	GC-FID	↑Lactic acid ↑Acetic acid ↑Propionic acid ↑Total SCFA	[180]
Plant	High-amylose maize (HAMS-D)	Human fecal fermentation	*In vitro*	GC-FID	↑Butyrate ↑Acetate ↑Propionate ↑Total SCFA	[88]
Algae	*Enteromorpha compressa* *Sargassum wightii* *Acanthophora spicifera*	Probiotic culture broth	*In vitro*	RPLC-RID	↑Butyric acid ↑Propionic acid ↑Acetic acid	[181]
Plant	*Dendrobium huoshanense* (GXG)	Mice luminal contents	*In vivo*	GC-FID	↑Acetic acid ↑Propionic acid ↑*n*-Butyric acid ↑n-Valeric acid ↑i-butyric acid ↑i-valeric acid	[182]
Plant	*Rosa roxburghii* fruit (RTFP-3)	Human fecal fermentation	*In vitro*	GC-MS	↑Acetic acid ↑Propionic acid Plant ↑Butyric acid ↑n-Butyric acid ↑i-Valeric acid ↑n-Valeric acid ↑Total SCFAs	[183]
Algae	*Gracilaria Lemaneiformis* (SP)	Human fecal fermentation	*In vitro*	GC	↑Acetic acid ↑Propionic acid ↑*n-*Butyric acid ↑Isobutyric acid ↑*n-*Valeric acid ↑Isovaleric acid	[163]
Plant	Softwood hemicellulose (PP)	Mice fecal Fermentation	*In vivo*	HPLC-UV	↑Acetic acid ↑Propionic acid ↓Formic acid ↑Butyric acid	[184]
Plant	Aloe Pochapski et al. (2021)	Mice fecal Fermentation	*In vivo*	GC-MS	↑Acetic acid ↑Propionic acid ↑*n-*Butyric acid ↑Isobutyric acid ↑*n-*Valeric acid ↑Isovaleric acid ↑Total SCFAs	[185]
Plant	Flaxseed (FSP)	Human fecal fermentation	*In vitro*	GC-FID	↑Propionic acid ↑*n*-Butyric acid ↑Acetic acid ↑n-Valeric acid ↑i-Valeric acid ↑i-Butyric acid	[186]
Plant	Tamarind Seed (TSP)	Human fecal fermentation	*In vitro*	GC-MS	↑Acetic acid ↑Propionic acid ↑i-Butyric acid ↑*n*-Butyric acid ↑i-Valeric acid ↑n-Valeric acid	[187]
Plant	Carrot (RG-1)	Human fecal fermentation	*In vitro*	GC-FID	↑Acetate ↑Lactate ↑Propionate ↑n-Butyrate ↑n-valerate ↑i-Butyrate ↑i-Valerate	[188]
Plant	Okra (OPP-D)	MRS Broth fermentation by probiotics	*In vitro*	GC-FID	↑Total SCFA	[189]
Plant	*Gracilaria lemaneiformis (GLP)*	Human fecal fermentation	*In vitro*	GC-FID	↑Acetic acid ↑Propionic acid ↑Iso-Butyric acid ↑Butyric acid ↑Isovaleric acid ↑Valeric acid	[190]
Plant	Quinoa (QPS)	Human fecal fermentation	*In vitro*	GC-MS	↑Butyric acid ↑Propionic acid ↑Valeric acid ↑Acetic acid ↑i-Valeric acid ↑i-Butyric acid	[191]
Bacteria	Exopolysaccharides from *Lactobacillus rhamnosus* ZFM231	Human fecal fermentation	*In vitro*	GC-FID	↑Acetic acid ↑Propionic acid ↑Butyric acid ↑i-Butyric acid ↑Valeric acid ↑i-Valeric acid	[167]
Plant	Okra (OPP-D)	Human fecal fermentation	*In vitro*	GC-FID	↑Acetic acid ↑Propionic acid ↑Butyric acid ↑n-Butyric acid ↑i-Valeric acid ↑n-Valeric acid ↑Total SCFA	[192]
Arthropods	Chito-oligosaccharides (COS2500)	Mice fecal fermentation	*In vivo*	GC	↑Acetic acid ↑Propionic acid ↑Butyric acid	[137]
Plant	*Dendrobium officinale* leaf (DOLP)	Mice fecal fermentation	*In vivo*	GC-FID	↑Acetic acid ↑Propionic acid ↑Butyric acid ↑Valeric acid ↑Total SCFAs	[193]
Plant	*Fortunella margarita* (FMPS)	Mice fecal fermentation	*In vitro*	HPLC-UV	↑Butyric acid ↑Acetic acid ↑Propionic acid ↑Lactic acid	[194]
Plant	*Ziziphus Jujuba cv. Pozao (JPS)*	Human fecal fermentation	*In vitro*	GC	↑Acetic acid ↑Propionic acid ↑n-Butyric acid ↑Total SCFA	[15]
Plant	Sweet tea leaves (P-DM)	MRS Broth fermentation by probiotics	*In vitro*	GC-FID	↑Total SCFA	[195]
Bacteria	Exopolysaccharides from *Lactobacillus rhamnosus* ZFM231	Mice fecal fermentation	*In vivo*	GC-FID	↑Acetic acid ↑Propionic acid ↑Butyric acid	[169]
Plant	Date flesh and seed (DFP; DSP)	MRS Broth fermentation by probiotics	*In vitro*	HPLC	↑Lactic acid ↑Acetic acid ↑Propionic acid ↑*n-*Butyric acid ↑Total SCFAs	[196]
Bacteria	Exopolysaccharides from *Lactobacillus delbrueckii* (EPS-LB3) *Lacticaseibacillus rhamnosus (EPS-MLB3)*	Human fecal fermentation	*In vitro*	HPLC-PDA	↑Acetic acid ↑Propionic acid ↑Butyric acid	[197]
Mast cells	Heparin	Human fecal fermentation	*In vitro*	GC-FID	↑Acetate ↑Butyrate ↑Propionate	[166]
Bacteria	Exopolysaccharides from *Enterococcus faecium* (EPS-LB3) *Streptococcus thermophilus (EPS-MLB10)*	Human fecal fermentation	*In vitro*	HPLC-PDA	↑Acetic acid ↑Propionic acid ↑Butyric acid	[198]
Plant	Date seed (MPS; UPS)	Human fecal fermentation	*In vitro*	HPLC-PDA	↑Acetic acid ↑Propionic acid ↑Butyric acid	Jayasree Subhash et al. [11,199]
Plant	Date Pomace (MPS; UPS)	Human fecal fermentation	*In vitro*	HPLC-PDA	↑Acetic acid ↑Propionic acid ↑Butyric acid	[200]; Bamigbade et al. [10]
Plant	Carob pods (MPS-1)	Human fecal fermentation	*In vitro*	HPLC-PDA	↑Acetic acid ↑Propionic acid ↑Butyric acid	[9]
Plant	Mango seed kernel (MSKP)	Human fecal fermentation	*In vitro*	HPLC-PDA	↑Acetic acid ↑Propionic acid ↑Butyric acid	[155]

↓-Decrease, ↑-Increase, HPLC-PDA- GC-FID-Gas Chromatography equipped with Flame Ionization Detector, RPLC-RID-Reverse Phase Liquid Chromatography equipped with Refractive Index Detector, GC-MS-Gas Chromatography equipped with Mass Spectrometry, GC-Gas Chromatography, HPLC-High Performance Liquid Chromatography equipped with Ultraviolet Detector, HPLC-UV-High Performance Liquid Chromatography, HPLC-PDA-High Performance Liquid Chromatography equipped with Photo Diode Array Detector.

Dietary pectin also influences gut microbiota composition and diversity. Multiple in vitro and *in vivo* studies report that pectins increase *Lactobacilli*, *Bacteroides*, and *Prevotella* populations [[201], [202], [203], [204]]. However, some *in vivo* studies noted inconsistent effects, with both increases and decreases in *Lactobacillus* and *Bacteroides* depending on study conditions [205,206].

Acacia gum (AG), a branched polysaccharide composed mainly of arabinose and galactose, has a long tradition of medicinal use in African and Middle Eastern cultures for treating gastrointestinal disorders. Fermentation studies showed that AG significantly promotes *Bifidobacteria* proliferation, comparable to fructo-oligosaccharides (FOS), and inhibits *Clostridium histolyticum*, commonly associated with dysbiosis ([175]; Tiencheu et al.). In constipated mice, treatment with 100 mg/kg/day of nano-crystalline cellulose (NCC) increased the relative abundances of *Lactobacillus* and *Anaerotruncus* by more than 177%, restoring them to normal levels. The relative abundance of Prevotellaceae*_UCG-001* increased by 40.9% as well, indicating NCC's role in modulating SCFAs levels and influencing the farnesoid X receptor (FXR) pathways [72].

Dietary cellulose is linked to gut homeostasis by expanding symbiont populations and enhancing beneficial metabolites. Bacteroidetes accounted for roughly 50% and 40% of gut microbiota in low- and high-cellulose diet-fed mice, respectively, with *Bacteroides* as the dominant genus in both groups. Notably, *Akkermansia muciniphila* abundance was significantly higher in high-cellulose-fed mice, confirmed by metagenomic sequencing [69]. Richness and diversity metrics, such as OTUs and Shannon index, were comparable between the two diet groups. In another study, constipation reduced *Bacteroides*, *Alloprevotella*, Ruminococcaceae*_UCG-014*, and *Ruminiclostridium_9* populations. However, NCC supplementation increased *Bacteroides* abundance by 21.7%, restoring it to near-normal levels [72].

Targeting the gut microbiota through probiotics, prebiotics, and dietary interventions has emerged as an effective strategy to treat various metabolic and medical conditions. Gut microbiota and their metabolites influence host immunity, inflammation, and metabolic regulation (Sedigheh Taghinezhad-S, 2020). Extensive evidence suggests polysaccharides modulate the gut microbiota to ameliorate metabolic diseases such as obesity, Type 2 diabetes mellitus, metabolic dysfunction-associated fatty liver disease (MAFLD), colorectal cancer, and irritable bowel syndrome (Hiel et al.).

Dysbiosis across different gut microflora groups plays a significant role in disease development. Understanding the microbial functions associated with health conditions is crucial for preventing dysbiosis. For instance, *Eubacterium* and *Bacteroides* regulate cholesterol by converting it into bile acids and coprostanol, while *Lactobacillus* and *Bifidobacteria* strains promote bile acid deconjugation and fecal cholesterol excretion, helping to lower serum cholesterol levels.

The anti-obesity effects of *Ficus carica Linn.* polysaccharide (FCPS), peach gum polysaccharide (PGPS), and their combination (FCPG) were evaluated by monitoring changes in gut microbiota and SCFAs release in vitro [207]. Both FCPS and FCPG exhibited strong abilities to produce SCFAs, lower pH, and generate gas, while PGPS showed limited effects. The total SCFAs content was highest in the FCPS group, with elevated acetic acid levels, followed by the combination group. Propionic acid levels were notably higher in the FCPG group. Initially, Firmicutes were dominant, but after 24 h, *Bacteroides* increased and Firmicutes decreased, leading to a lower Firmicutes/Bacteroidetes (F/B) ratio, a marker associated with anti-obesity effects.

Different dietary fiber groups and their gut microbiota-mediated impacts on metabolic disorders were summarized by Ref. [208]. Ten FDA-approved natural, synthetic, and isolated non-digestible carbohydrates were evaluated for their anti-obesity effects in obese rat models. Supplementation with β-glucan, arabinoxylan, xanthan gum, guar gum, apple pectin, carrageenan, inulin, and xylans significantly reduced body weight and dyslipidemia. However, glucomannan and arabinogalactan showed no significant effects. Major fibers enriched beneficial bacteria such as *Akkermansia*, *Butyricimonas*, *Oscillospira*, *Ruminococcus*, and *Prevotella*, correlated with improved cholesterol profiles and metabolic health.

Suppression of high-fat diet (HFD)-induced obesity and inflammation was also observed with Polysaccharides from sporoderm-broken spores of *Ganoderma lucidum* (BSGLP) [209]. BSGLP supplementation improved GPR43 expression, reduced total and LDL cholesterol levels, and inhibited liver steatosis. Supplementation also decreased the F/B ratio, enhanced acetic acid production, and increased beneficial gut bacteria, such as *Bifidobacterium*, *Allobaculum*, and *Christensenellaceae_R-7_*, which were absent in untreated obese mice.

Similarly, Polysaccharides from *Moringa oleifera* (MOP) prevented obesity and lipid accumulation in HFD-induced mice. MOP supplementation reshaped the gut microbiota, increasing the abundance of *Bacteroides*, *norank_f_*Ruminococcaceae, and *Oscillibacter*, while reducing *Blautia*, *Alistipes*, and *Tyzzerella* taxa, which are associated with obesity [210]. These microbial changes correlated with improved serum lipid profiles, reduced insulin resistance, decreased pro-inflammatory cytokine levels, and regulated lipid and bile acid metabolism genes.

Comprehensive reviews by Xiao et al. [211,212] discussed the impact of non-starch Polysaccharides on gut microbiota-modulated anti-Type 2 Diabetes Mellitus (T2DM) activity and the brain-gut axis in neurodegenerative disorders, respectively. Using a T2DM rat model, She et al. [213] demonstrated that *Grifola frondosa* Polysaccharides prevented hyperglycemia, improved insulin sensitivity, repaired intestinal mucosal barrier damage, and alleviated skeletal muscle atrophy.

Polysaccharides from *Phellinus linteus* (PLP) also improved Bacteroidetes abundance, decreased Firmicutes levels, and alleviated insulin resistance in T2DM rats [214]. For metabolic dysfunction-associated fatty liver disease (MAFLD), supplementation with CQ-1, a water-soluble Polysaccharides extracted from *Acanthopanax senticosus*, reduced pro-inflammatory cytokines, triglycerides, LDL-cholesterol, and enhanced liver antioxidant enzyme activity [215]. CQ-1 supplementation also increased beneficial bacteria such as *Bifidobacteria* and *Lactobacillus*, raised SCFAs levels (acetic, propionic, isobutyric, n-butyric, and n-valeric acids), and suppressed the growth of *Unclassified Desulfovibrionaceae*, implicated in MAFLD pathogenesis.

Table 3 summarizes different polysaccharides structures and their associated health effects mediated by modulation of the gut microbiota.

**Table 3:** Polysaccharide structural features and their correlated health effects via gut microbiota modulation.

Gut microbiome/group	Origin and type	Polysaccharide	Health Impacts associated with gut microbes	References
↑*Lactobacillus* and *Bacteroides,* and ↓ *Staphylococcus*.	Neutral plant polysaccharide	Turmeric polysaccharides (TPs)	TPs treatment reversed the abnormal metabolic function of intestinal flora, such as biosynthesis and gluconate metabolism in cyclophosphamide-treated mice.	[216]
↑Firmicutes, and ↓ Proteobacteria	Plant Heteropolysaccharide	*Schisandra chinensis* (Turcz.) Baill polysaccharide (SACP)	SACP alleviated Dextran Sulfate Sodium Salt (DSS) induced ulcerative colitis by regulating the diversity and composition of intestinal microbiota in treated mice.	[217]
↑Firmicutes and ↓ Proteobacteria.	Homogeneous acidic fucosan polysaccharide from plants	*Fucus vesiculosus* Polysaccharides (FVP-7 A)	FVP-7 showed improved potential to increase the richness of intestinal microbiota in diabetics	[218]
↑Bacteroidetes and ↓ Firmicutes	Sulfated Plant Polysaccharide	*Sargassum fusiforme* (SFSP)	SFSP showed potential to downregulate the contents of trimethylamine, piperidone, secondary bile acid, ↑ nicotinic acid, pantothenic acid, etc.	[219]
↓Bacteroidetes and Proteobacteria	Pectin-like plant polysaccharide	Chestnut Polysaccharides (CPs)	CPs restored impaired spermatogenesis in busulfan-treated mice by altering gut microbiota, as evidenced by the steroid hormone biosynthesis in KEGG level 3 pathway analysis.	[220]
↓Bacteroidetes and ↑ Firmicutes	Microbial exopolysaccharides from *G. lucidum* mycelium	*Ganoderma lucidum* strain S_3_(GLP S_3_) Polysaccharides	GLP S_3_ displayed downregulation of the pathways for pancreatic Hyproxyline, TNF-α, and IFN- ɣ, restoring the secretion of pancreatic lipase and AMS, inhibiting chronic pancreatitis in diethyldithiocarbamate (DDC) induced mice.	[221]
↓Bacteroidetes, Proteobacteria*,* and sulfate-reducing bacteria	Compound Polysaccharides (CP) from plants	Combination of Chinese Yam (*Dioscorea opposita Thunb*.) polysaccharides (CYP) and inulin	CP effectively reversed 2,4,6-trinitrobenzenesulfonic acid (TNBS)-induced colitis in rats via restoration of dysregulated gut microflora, including improvement on basic metabolism and a reduction in pathogenesis processes.	[222]
↓ Firmicutes to Bacteroidetes abundance ratio	Homogeneous plant polysaccharide	*Lycium barbarum* polysaccharides (LBP-W)	LBP-W supplements could effectively alleviate hyperglycemia and hyperlipidemia in high-fat diet (HFD)-induced mice.	[223]
Restoration of the abundance of *Bacteroides* and Firmicutes	Homogeneous plant polysaccharide	*Sagittaria sagittifolia L.* polysaccharides (POLYSACCHARIDESP-1)	POLYSACCHARIDESP-1 treatment upregulated the expression levels of claudin-1, occludin, and ZO-1, inhibited protein phosphorylation of molecules in MAPK and NF-κB signaling pathways, and mitigated colitis symptoms in DSS-treated mice.	[224]
↓Firmicutes and Proteobacteria*,* ↑ Bacteroidota	Neutral plant polysaccharide	Alhagi honey polysaccharide (AHPN50-1a)	Supplementation with AHPN50-1a downregulated the expression of pro-inflammatory cytokines (IL-1β, IL-6, TNF-α) in a murine model of mice with DSS-induced colitis	[225]
↓*Bacteroides*, *Intestinimonas*, *Parabacteroides*, and restoration of F/B ratio	α-acidic pyran heteropolysaccharide from plant	*Phyllanthus emblica* L. polysaccharides (PEPs)	PEP supplementation downregulated Akt, p38, ERK, and JNK phosphorylation, which are indicators of colitis aggravation in 2,4,6-trinitrobenzene sulfonic acid (TNBS) treated rats.	[226]
↓Firmicutes and ↑ proliferation of Bacteroidetes	Plant heteropolysaccharide	Polysaccharides from *Phellinus linteus* (PLP)	PLP effectively modulates gut microbiota and bile acids metabolism by stimulating Glucagon-like Peptide-1, improving insulin release, and alleviating Type 2 Diabetes Mellitus in rat model.	[214] [37]
↓relative ratio of Firmicutes to Bacteroidetes	Heteropolysaccharide from plant residue	*Laiyang* pear residue polysaccharide (LPP)	LPP mitigated DSS-induced acute colitis in mice by upregulating intestinal barrier integrity and TJ protein expressions and suppressing inflammation.	[227]
↑Bacteroidota and ↓Proteobacteria	Homogeneous polysaccharide from plant	*Polygonatum cyrtonema* polysaccharides (PCP)	PCP restored IgA, ZO-1, Occludin, and MUC2 expression, alleviating colitis in DSS-induced colitis mouse model	[228]
↑*Lactobacillus* and *Bifidobacterium*, ↓ *Desulfovibrio*	Heterogeneous plant polysaccharide	*Dendrobium officinale* leaf polysaccharide (DOLP)	DOLP improved the release of immune-related cytokines (TNF-α, INF-γ, and IL-6) in immunosuppressed mice, exerting immune modulating effects	[229]
↑*Bacteroides* and Firmicutes	Purified Plant polysaccharide	Deglet Noor date polysaccharides (DP)	DP alleviated amoxicillin-induced diarrhea, reversed the antibiotic-induced dysbiosis, and improved Mucin-2 expression in treated mice.	[230]
↓Firmicutes*/*Bacteroidota (F/B) ratio	Plant heteropolysaccharide	*Dendrobium officinale* Polysaccharide (DOP)	DOP significantly reduced serum lipid levels and inflammatory factors, alleviating High-Fat Diet-Induced Atherosclerosis in mice	[231]
↑*Blautia* and *Bifidobacterium*	Purified Plant polysaccharide	*Brassica rapa L.* Polysaccharide (BP)	BP aided in tumor weight reduction and downregulated the expression of Ki67 protein, promoting tumor cell apoptosis in Lewis Lung Cancer tumor-bearing mice	[232]
↓Firmicutes*/*Bacteroidetes ratio, ↑ *Akkermansia*	Purified Plant polysaccharide	*Crataegus pinnatifida* polysaccharide (CPP)	CPP improved free fatty acid (FFA)-induced lipid accumulation in HepG2 cells, activating fatty acid oxidation genes against non-alcoholic fatty liver disease (NAFLD) in mice	[233]
↑Bacteroidetes, *Akkermansia,* and *Blautia*	Homopolysaccharide from plant	*Aloe barbadensis Miller* polysaccharides Pochapski et al. [234]	Aps showed effective activation of 6-pyruvoyltetrahydropterin synthase in the folate biosynthesis metabolism pathway and inhibition of the phosphotransferase system in KEGG analysis to alleviate weight loss, colonic inflammation, and subacute ulcerative colitis (SUC) in mice.	[235]
↑Actinobacteria and *Bifidobacterium*	Fructans from plants	Inulin Type Fructans (ITF)	ITF supplementation induced significant weight loss, improved BMI, diastolic blood pressure, AST, insulinemia, and reduced fasting insulin levels compared to Metformin-treated patients	[236]
*↓*Firmicutes*/*Bacteroidetes ratio	Plant Oligosaccharide	Crude gac aril (*Momordica cochinchinensis*) polysaccharides (GAP)	GAP inhibited body weight gain, hyperlipidemia, and improved insulin sensitivity, impaired glucose tolerance in HFD-induced obese mice.	[237]
*↑*Firmicutes*,* Bacteroidota*, Patescibacteria* and *Desulfobacterota*	Purified Plant polysaccharide	Astragalus polysaccharides Pochapski et al. [234]	APS mitigates chemotherapy-induced (5-fluorouracil (5-Fu)) immune dysfunction in mice model by preventing colon shortening, upregulating the expression of intestinal barrier proteins, and maintaining linoleic acid (LA) and α-linolenic acid balance in polyunsaturated fatty acid (PUFA) metabolism.	[238]
Regulated the dysbiosis of *Akkermansia*, *Lactobacillus*, and *Bacteroides*	Purified Plant polysaccharide	*Gastrodia elata* polysaccharide (GEP)	GEP modulated the motor dysfunction, α-synuclein secretion, loss of dopaminergic neurons, and inactivation of TLR4/NF-κB pathway in the brain of 1-methyl-4-phenyl-1,2,3,6-tetrahydropyridine (MPTP)-induced Parkinson's disease in mice	[239]
*↑*Firmicutes	Selenized Plant Polysaccharide	Sweet corn cob selenium polysaccharide (SeSCP)	SeSCP efficiently reduced insulin resistance and blood glucose levels, altering the LPS/IκBα/NFκB pathway, improving lipid and carbohydrate metabolism in KEGG pathways in streptozotocin (STZ) induced type 2 diabetes mellitus mice	[240]
*↑Muribaculaceae*, *Akkermansia*, ↓*Escherichia-Shigella*, and *Proteus*	Fungal polysaccharide	*Agaricus bisporus* polysaccharides (ABPs)	Lower molecular weight purified fraction ABP-2, showcased enriched bile and tryptophan metabolism, and prevention against body weight loss, colon atrophy, offering improved therapeutic potential against dextran sodium sulfate (DSS)-induced colitis in mice	[241]

↓-Decrease, ↑-Increase, polysaccharides-Polysaccharides.

## 6 Polysaccharides in real food systems

The relevance of real food systems is an immediate consequence of the structure-function relations described in the preceding sections. The polysaccharide's modulation of the gut microbiota is not only dependent on its botanical/biological origins but also on its size, pattern of linkages, degree of branching, charge, and supramolecular organization because these factors will influence its bioaccessibility during digestion, its interactions in the food matrix, and its bioavailability for fermentation in the colon. Thus, application and processing can be viewed as an extension of mechanistic concepts: formulation can provide protection or concealment of motifs available for fermentation, while processing can provide conservation, unmasking, or modification of structural features controlling short-chain fatty acid production, cross-feeding interactions, and health effects. From this viewpoint, real food systems do not exist in isolation from mechanistic concepts; rather, they represent the context in which mechanistic potential is expressed or dissipated.

### 6.1 Application of dietary polysaccharides in real food systems

Within food formulations, these biopolymers are widely used because of their biocompatibility, biodegradability, and broad structural diversity. Mostly, they are used as thickeners, stabilizers, emulsifiers, gelling agents, and water retention agents to enhance the rheological properties, texture, and physicochemical stability of a wide variety of food products. Moreover, Polysaccharides are used as fat replacers, prebiotics, dietary fibers, and gastroprotectants to enrich the nutritional profile and functional health value of food products [242]. Among these, starch derivatives are the most commonly used Polysaccharides, followed by galactomannans, carrageenans, agars, alginates, pectins, cellulose derivatives, and xanthan gum [[242], [243], [244]].

Beyond texture modification, these polymers also serve as encapsulation matrices for bioactive compounds such as probiotics, flavoring agents, and antimicrobials. Research evidence indicates that polymers of polysaccharide origin such as alginate, chitosan, starch, pectin, and carrageenan can protect labile bioactive agents by acting as protective encapsulation matrices in functional food systems to enhance the stability and shelf life of these agents [244,245].

In the field of food packaging, polysaccharides are commonly used as biodegradable and edible coating agents to extend product shelf life while maintaining product freshness. Polysaccharides can be engineered to produce bioactive films with antimicrobial activity to meet the demand for green food packaging [246]. Additionally, Polysaccharides are used to produce meat and fat substitutes through protein-polysaccharide conjugates. These conjugates facilitate the creation of anisotropic textures, fibrous textures, moisture retention, and flavor perception within food systems. These protein-polysaccharide conjugates are crucial for the production of plant-based meat analogues and other novel structured foods [247].

Chemically or physically modified polysaccharides extend potential applications within food systems by enhancing inherent properties such as solubility, rheological properties, emulsifying properties, and biological activity. Structurally modified Polysaccharides are also used to support emerging technologies for 3D printing within food systems. Despite these benefits, challenges persist with regards to incorporating these modified polysaccharides into complex food systems [248].

**Fig. 11:**
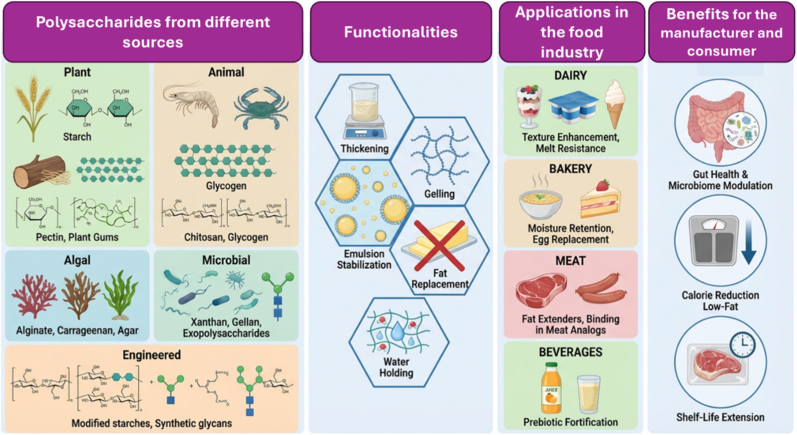
Polysaccharide applications in real food systems (The figure was generated with the assistance of https://scispace.com based on author-provided input and subsequently reviewed and edited for scientific accuracy.).

Among the marine-derived Polysaccharides are alginate, agar, and carrageenan, which have received considerable interest in the dairy industry. This is owing to the gelling potential of these materials, which can be either ionically cross-linked or thermoreversible gels, making them extremely valuable in the production of restructured foods and encapsulation systems [249]. From the above examples, it can be concluded that the successful application of Polysaccharides from molecular structure to food systems requires an understanding of formulation conditions, interactions between ingredients, and the desired health outcomes. This versatility of Polysaccharides makes them key players in the production of fiber-enriched, microbiota-oriented, and clean label food products. Fig. 11 shows the different sources of Polysaccharides, their major techno-functional roles in food systems, major food products, and the health and preservation benefits they impart to the products. Table 4 provides an overview of selected polysaccharides categorized by source, highlighting their primary functional roles in food systems, typical applications across product categories, and resulting technological and health-related outcomes.

**Table 4:** Polysaccharide applications in real food systems.

Polysaccharide Type/Source	Key Functional Roles	Food Applications	Functional Outcomes
**Starch (cereals, tubers)**	Thickener, gelling agent, texture modifier	Bakery products, sauces, noodles	Improved viscosity, gelation, and mouthfeel
**Pectin (fruits)**	Gelling, stabilizing, fat replacer	Jams, jellies, low-fat spreads	Stable gels, reduced syneresis, improved spreadability
**Chitosan (crustacean shells)**	Film-forming, antimicrobial, fat barrier	Edible coatings, meat preservation, packaging films	Extended shelf life, reduced microbial growth
**Xanthan Gum (microbial)**	Thickening, suspension stabilizer	Salad dressings, beverages, gluten-free bakery	Stable viscosity, suspension of particulates
**Guar Gum (legume seeds)**	Thickening, water retention	Ice cream, sauces, gluten-free bread	Creamy texture, reduced ice crystal growth
**Alginate (seaweed)**	Gelation, encapsulation	Reformed meat, dairy desserts, probiotic encapsulation	Controlled release, stable gels
**Carrageenan (seaweed)**	Gelling, stabilizing	Dairy products, plant-based milks	Improved mouthfeel, suspension stability
**Cellulose & Hemicellulose (plant cell walls)**	Dietary fiber, texture modifier	Bakery, meat analogues, fiber-enriched foods	Increased fiber content, improved structure
**Fructo-, Galacto-, Xylo-oligosaccharides (date pomace)**	Prebiotic, functional ingredient	Yogurt, infant formula, functional beverages	Gut microbiota modulation, improved digestion
**β-Glucans (cereals, fungi)**	Cholesterol-lowering, immune modulation	Functional breads, beverages, supplements	Health claims support, viscosity enhancement

This table has been summarized from Ref. [[250], [251], [252], [253], [254], [255]]

### 6.2 Processing aspects of polysaccharides in food systems

The processing of Polysaccharides in food systems may include different physical, chemical, and enzymatic methods that can affect the molecular structure of these biopolymers, their physicochemical properties, and functional activities. The changes in the structure of these biopolymers can affect their interactions with proteins, lipids, and other components of the matrix, which can influence the texture, stability, shelf life, and nutritional value of the final product [256,257].

Processing technologies of polysaccharides can be divided into conventional technologies and newly developed non-thermal technologies. Conventional processing technologies, including dehydration and thermal treatment, significantly affect the structural configuration and functional properties of Polysaccharides, thereby influencing their functionality and nutritional quality. For example, thermal treatment can trigger the Maillard reaction, causing the formation of undesirable sensory properties of the final product. Hence, the application of suitable drying technologies is crucial during the processing of Polysaccharides, considering their application in the food industry. In contrast, the application of newly developed technologies, including ultrasonication, high-pressure treatment, and microwaving, can result in the controlled depolymerization of the polysaccharide molecules. This, in turn, can significantly affect the rheological properties of the polysaccharide. Noteworthy, the application of high-pressure treatment and PEF treatment can maintain the molecular structure of the polysaccharide, thereby retaining the nutritional properties of the polysaccharide. In contrast, ultrasonication treatment can result in the scission of the polysaccharide molecules, thereby modifying the properties of the polysaccharide, considering their application in the food industry [258,259].

In recent times, there has been a growing interest in modifying Polysaccharides chemically and in terms of nanoengineering to make these materials more efficient in terms of physicochemical attributes. To this end, various approaches such as esterification, etherification, graft copolymerization, crosslinking, and sulfation have been explored to make Polysaccharides more efficient in terms of solubility, mechanical strength, and bioactivity. At the same time, approaches in polysaccharides nanoengineering, including nanocellulose, starch nanocrystals, chitosan nanoparticles, and polysaccharides-based nanocomposites, have been explored to make Polysaccharides more efficient in terms of morphology, surface activity, and interfacial activity [[260], [261], [262]]. Despite these recent advances in polysaccharides-based processing strategies, there are challenges in adopting these approaches at larger scales to make these approaches cost-effective and commercially viable. It is important to understand that food systems are complex in terms of structure-function relationship. It is important to understand these complex relationships to assess how polysaccharides-based ingredients would behave in food systems. At the same time, it is important to assess safety and toxicity profiles to make polysaccharides-based ingredients more efficient in food systems [248]. To make polysaccharides-based ingredients more efficient in terms of broadening their versatility in food systems, recent advances in polysaccharides-based processing strategies are important. Table 5 summarizes conventional, emerging, and green processing techniques used to modify polysaccharides, detailing their mechanisms of action, effects on molecular structure and functionality, and technological implications in food systems. Therefore, processing should be evaluated not only for techno-functional performance, but also for its capacity to preserve the structural determinants of microbial utilization and health-related functionality.

**Table 5:** Polysaccharide processing aspects in real food systems.

Processing Method	Mechanism/Effect on Polysaccharide	Impact on Structure & Functionality	Functional Outcomes in Foods	References
Thermal Processing (cooking, baking, extrusion)	Depolymerization, Maillard reaction, gelatinization	Reduced molecular weight, altered gelation, viscosity changes	Modified texture, possible nutrient loss	[258,263]
Drying/Dehydration	Water removal, structural collapse	Reduced solubility, altered rehydration	Shelf-stable powders, altered mouthfeel	[256]
High-Pressure Processing (HPP)	Conformational changes without major degradation	Enhanced gelation, increased resistant starch	Improved texture, bioactivity retention	[248,264]
Cold Plasma (CP)	Surface modification, cross-linking	Improved rheology, antioxidant content	Enhanced stability, functional enrichment	[248,265,266]
Ultrasound Treatment	Cavitation-induced chain scission	Increased solubility, extraction yield	Higher bioactivity, improved dispersion, gut microbiota modulation	[267]
Pulsed Electric Field (PEF)	Electroporation, structural loosening	Minimal degradation, improved extraction	Retained bioactivity, better functional integration	[268,269]
Chemical Modification (carboxymethylation, sulfation, oxidation)	Introduction of functional groups, cross-linking	Increased solubility, stability, bioactive delivery	Expanded application range, improved performance	[251,270,271]
Nanotechnology Integration	Nano-encapsulation, particle size reduction	Targeted delivery, controlled release	Enhanced bioavailability, gut microbiota modulation	[250,272,273]
Multi-Component Matrix Design	Blending with proteins/lipids	Synergistic texture, stability	Stable emulsions, improved mouthfeel	[274,275]
Green Processing Dynamic High-Pressure Microfluidization	Mechanical disruption, enhanced dispersion	Higher extraction yield, bioactivity	Sustainable processing, improved functional properties	[248,266]

## 7 Challenges and limitations

### 7.1 Extraction and purification challenges

The methods used for extracting and purifying Polysaccharides from natural sources significantly influence their potency in the gut microbiota modulation. Many researchers have studied how Polysaccharides isolated using different methods affect gut microbiota composition [9], [11], [276]. Structural features such as molecular weight, glycosidic linkages, and monosaccharide composition show strong correlations with their modulatory effects, and these features can vary depending on the extraction and purification techniques employed [277].

Natural polysaccharides sources are structurally complex, often containing proteins, polyphenols, and other macromolecules, making the isolation of pure Polysaccharides challenging [278]. High-purity algal Polysaccharides demonstrate enhanced biological functionalities, including antiviral, anticoagulant, immunomodulatory, antioxidant properties, and gut microbiota modulation, which are critical for pharmaceutical applications [279]. However, the production of purified algal Polysaccharides remains costly due to the complex and expensive extraction protocols involved [280].

Extraction and purification procedures aim to preserve the inherent properties of Polysaccharides; thus, protocols should be optimized based on the intended application [48]. Researchers have used a variety of techniques to isolate Polysaccharides, including conventional hot water extraction, ultrasound-assisted extraction (UAE), microwave-assisted extraction (MAE), ultrasound-microwave-assisted extraction (UMAE), ultrahigh pressure-assisted extraction (UPAE), and enzyme-assisted extraction (EAE) [9] [276] [281]. Novel methods such as subcritical water extraction (SWE), pulsed electric field-assisted extraction (PEFAE), two-phase aqueous extraction (ATPE), and integrated techniques like nanoparticle technology, homogenization extraction, vacuum extraction, and electrolytic oxidation water extraction are also being explored [48,282]. Each extraction approach offers unique advantages and challenges in terms of cost, material complexity, time, energy consumption, environmental impact, therapeutic potential, and extraction efficiency [282].

For instance, Polysaccharides extracted from loquat (*Eriobotrya japonica*) leaves using UMAE demonstrated higher prebiotic potential due to their lower molecular weight and viscosity [283]. However, scalability and cost issues associated with UAE, MAE, and UMAE limit their broader application [283]. Moreover, extraction conditions can cause excessive depolymerization, altering polysaccharides fermentation profiles by gut microbiota. For example, Polysaccharides extracted from *Porphyra haitanensis* via UMAE showed that extraction conditions significantly influenced molecular weight distribution, impacting digestibility and fermentation during in vitro simulations [284].

In some cases, glycosidic bond modifications during extraction may reduce digestibility, enhancing the ability of Polysaccharides to reach the colon intact for microbial fermentation [285]. However, the loss of side chains or branching structures can decrease prebiotic potential, as some gut microbes depend on specific structural features [11].

Hyaluronic acid (HA) can also be broken down by gut microbiota, with variations in metabolite production depending on the degree of depolymerization [54]. Similarly, although deep eutectic solvents (DES) are promising for non-toxic, high-yield polysaccharides extraction, some DES formulations can cause depolymerization or chemical alterations, potentially impacting polysaccharides's bioactivity and subsequent SCFAs synthesis [286].

PS purification primarily aims to remove co-extracted substances such as proteins and polyphenols. However, purification can sometimes eliminate elements that enhance the overall bioactivity of Polysaccharides, including their ability to modulate gut microbiota. During in vitro simulated digestion and fecal fermentation, *Hizikia fusiforme* polysaccharides-polyphenol complexes and their pure components showed significant impacts on human gut microbiota, highlighting the importance of certain co-extracted compounds [287]. Some polysaccharides-polyphenol complexes exhibit enhanced biological activities related to gut health and general well-being, emphasizing the need to refine purification procedures to preserve synergistic bioactive substances [288].

The main aim of polysaccharides fractionation lies in the identification and characterization of particular structural components that play a crucial role in fermentation, bioavailability, and interactions with gut microbiota [267,289]. It has been noted that fractions obtained from a similar polysaccharides source may vary in their molecular weights, monosaccharide composition, ionic properties, and bioactivities, leading to varying effects on gut microbiota. For instance, in vitro fermentation studies conducted on Laminaria japonica polysaccharides showed that fractions with greater molecular weights and more complex monosaccharide compositions produced more amounts of SCFAs, showing greater regulation potential for specific metabolic pathways [290].

Different techniques employed for polysaccharides fractionation include solvent precipitation, ultrafiltration, and chromatography. However, some challenges may be posed by these techniques, which could ultimately affect the biological functionality of Polysaccharides and their capability to modulate gut microbiota. For instance, some fractionation techniques may cause changes in the structure that could affect the functionality of Polysaccharides. Moreover, contaminants that may be introduced during the process may pose a challenge in gut microbiota studies. This highlights the need to optimize the techniques employed for polysaccharides fractionation to ensure that Polysaccharides remain functional for effective modulation of gut microbiota [291].

### 7.2 Challenges in structural characterization and standardization

From an analytical perspective, polysaccharides present exceptional structural complexity because monosaccharide residues can be arranged through diverse glycosidic linkages, branching patterns, and molecular-weight distributions. The diversity can be attributed to the branching pattern, molecular weight distribution, type of glycosidic linkage, and monosaccharide composition. Polysaccharides can be classified as heteropolysaccharides if they are composed of more than one type of monosaccharide or homopolysaccharides if they are composed of a single type of monosaccharide residue [292]. The functional role of these complex molecules in interacting with the microbiota in the gastrointestinal system is significantly dependent on the complex structure of these molecules. The complex structure also accounts for the non-uniform distribution of molecular weights and variation in bioactivity. Hence, complete characterization of these complex molecules is required to understand their potential functional role [293,294].

The complete characterization of complex polysaccharide structures involves the application of a wide variety of analytical tools to determine the chemical composition, type of glycosidic linkage, molecular weights, and conformational features of these complex molecules. Gas chromatography (GC) and high-performance liquid chromatography (HPLC) are used to determine the type of monosaccharide residues present in these complex molecules along with their relative proportions [295]. The branching patterns of these complex molecules are also determined through the application of methylation and degradative methods. Fourier-transform infrared (FTIR) spectroscopy and nuclear magnetic resonance (NMR) spectroscopy are used to determine the type of functional groups present in these complex molecules [9]. The application of mass spectrometry (MS) techniques such as matrix-assisted laser desorption/ionization (MALDI-MS) and tandem MS enables the complete elucidation of complex polysaccharide structures [296]. Gel permeation chromatography (GPC) methods are also used to determine the molecular weights of these complex molecules [9] [11] [276].

However, limitations of these methodologies are associated with a comprehensive structural analysis. Although NMR is effective in structural analysis, a large amount of purified Polysaccharides is required to generate a robust signal. Impurities may also result in overlapping peaks, complicating the analysis [297]. In HPLC analysis, Polysaccharides that lack chromophores require derivatization, leading to increased complexity and accuracy issues. The derivatization agents, such as 1-phenyl-3-methyl-5-pyrazolone (PMP), may result in major peaks that mask monosaccharide peaks, especially in samples of low concentration [298].

In the molecular weight analysis of Polysaccharides by GPC, challenges may be encountered in the use of Pullulan standards. This is because Pullulan is a linear polysaccharides and may not be representative of branched Polysaccharides, potentially leading to incorrect molecular weight determinations of Polysaccharides. For example, branched Polysaccharides such as dextrans may not be adequately characterized by Pullulan standards due to differences in elution behavior [299].

The standardization of polysaccharides analytical techniques is essential to ensure accurate and reliable analysis of Polysaccharides, especially in prebiotic analysis. This is because the efficacy of prebiotics depends on polysaccharides structure; therefore, discrepancies in analysis should be minimized to ensure accurate and reliable findings and to support valid efficacy claims. Discrepancies in structural analysis may affect the reliability of prebiotic efficacy claims and potentially complicate comparisons across various studies [300].

### 7.3 Bioavailability and stability issues

PS degradation is also affected by various factors including chemical structure, branching degree, and molecular weight. For example, high amylose starches are more resistant to enzymatic degradation due to tightly packed starch granules [301]. On the contrary, highly branched polysaccharides, including amylopectin, are more prone to enzymatic degradation due to a highly branched structure. Some studies also suggest that linear Polysaccharides have a greater influence on gut microbiota modulation than branched Polysaccharides; for example, linear fructooligosaccharides have shown greater prebiotic potential by enhancing the growth of gut microbiota ([302]; Tiencheu et al.).

The structural integrity of Polysaccharides is important for determining the ability of these polysaccharides to modulate gut microbiota. For example, highly branched polysaccharides with high molecular weights are more resistant to enzymatic degradation in the upper GI tract; hence, these polysaccharides reach the large intestine intact for selective fermentation by beneficial gut microbiota, which produce SCFAs and modify gut microbiota composition. Conversely, Polysaccharides with low molecular weights are easily fermented, thereby producing a quick fermentation rate to produce gases and SCFAs while exerting a differential effect on gut microbiota composition [303]. Although low molecular weight polysaccharides are fermented quickly, high molecular weight polysaccharides have a longer residence time in the gut to exert a sustained effect on gut microbiota [304].

## 8 Clinical evidence and health outcomes of polysaccharide-gut microbiota interactions

### 8.1 Clinical studies and human intervention trials

Numerous studies have examined the ability of Polysaccharides to modulate gut microbiota both in vitro and *in vivo*. Several *in vivo* studies have demonstrated health-promoting benefits linked with polysaccharides-induced modulation of gut microbiota. However, to confirm gut microbiota-related health benefits, clinical and human intervention trials are essential.

The role of polysaccharides-based natural substance complexes in managing obesity and related metabolic disorders has been systematically reviewed [17,305,306]. In both human and animal studies, Policaptil Gel Retard® (PGR), an oral, polysaccharides-based macromolecule complex, showed significant reductions in body weight, blood glucose, insulin, and cholesterol levels. PGR also partially reversed the high-fat diet (HFD)-induced increase in Firmicutes abundance by efficiently sequestering macronutrients in the gut, acting against obesity.

Two parallel clinical trials evaluated the impact of snacks formulated with pea- and orange-derived Polysaccharides on gut microbiota and metabolic outcomes in adult female dizygotic twin pairs[173]. The participants included both concordant and discordant pairs for obesity. Results revealed significant increases in bacterial taxa associated with pectin-degrading specialists: *Monoglobus pectinilyticus* (pea fiber) and *Lachnospira pectinoschiza* (orange fiber). Additionally, the representation of key butyrate-producing genera such as *Lachnospira*, *Ruminococcus*, and *Faecalibacterium* increased, suggesting that dietary fibers from pea and orange promote beneficial microbial changes linked to gut health and obesity-related hormonal regulation.

Polysaccharides also help prevent conditions, such as constipation, colorectal cancer, and infections, by strengthening the intestinal barrier and supporting beneficial microbes. Two clinical studies (metagenomics and metabolomics-based) investigated the effects of sulfated Polysaccharides from *Ulva sp.* 84, xylorhamnoglucuronan (SXRG84), on metabolic markers, inflammation, and gut microbiota composition [307]. In the first study, 64 overweight participants received either 2 g/day or 4 g/day of SXRG84 for six weeks. In the second study, 64 participants underwent a placebo phase for six weeks, followed by 2 g/day of SXRG84 for another six weeks.

In study 1, a 2 g/day dose of SXRG84 led to significant reductions in non-HDL cholesterol, atherogenic index, and 2-h insulin levels. Significant shifts in gut microbiota were also observed, with increased levels of *Bifidobacteria*, *Akkermansia*, *Pseudobutyrivibrio*, and *Clostridium*, alongside decreased *Bilophila*. In contrast, study 2 showed an increase in *Fusicatenibacter* and *Parabacteroides* and a reduction in *Clostridium*, although metabolic marker improvements were not statistically significant.

Likewise, improvements in Bifidobacteria levels were noted in the assessment of the efficacy of Deshipu Stachyose Granules (DSG), an α-Galacto-Oligosaccharide derived from *Lycopus lucidus* Turcz, for its effects on the human gut microbiota and bowel functions [308]. In a study involving 100 healthy humans, the administration of DSG at a dose of 5 g/d for 14 days was noted to increase *Lactobacillus* and Bifidobacteria while decreasing *Clostridium perfringens*. In another study, DSG at a dose of 5 g/d for 30 days was noted to improve bowel movement frequency, stool softness, and ease of bowel movement in 103 constipated patients compared to a placebo group. These studies collectively suggest that DSG increases beneficial gut microbiota and relieves constipation.

A randomized, controlled, double-blind, parallel clinical trial evaluated the effect of a β-*d*-glucan-enriched (BGE) mixture derived from *Lentinula edodes* (shiitake mushrooms) on untreated mild hypercholesterolemia [309]. Conducted with 52 subjects aged 18 to 65 years, the study found no significant differences in lipid or cholesterol parameters between the BGE and placebo groups. However, in vitro and animal studies had previously indicated hypocholesterolemic effects of BGE. Interestingly, BGE administration appeared to modulate the colonic microbiota. An increased presence of Ruminococcaceae and *Bifidobacteria* was observed in BGE-treated subjects, and these bacteria were inversely correlated with cholesterol levels, suggesting a potential link between cholesterol metabolism and gut microbiota modulation via BGE.

Ongoing clinical trials are also examining the impact of *Lycium barbarum* Polysaccharides (LBP) on non-alcoholic fatty liver disease (NAFLD) through gut microbiota modulation [310]. In a randomized, double-blind, placebo-controlled study, the effects of LBP supplementation on intestinal permeability, gut microbial diversity and abundance, and alanine aminotransferase (ALT) concentration are being assessed in 50 NAFLD patients. Preclinical studies suggest that LBP may regulate NAFLD progression by improving gut microbial composition, leading to better liver function and gut health. Hsu and Cheng [311] provided a comprehensive report on several failed clinical trials related to *Ganoderma* extracts or Polysaccharides, underscoring the challenges and variability in translating polysaccharides-based interventions from animal models to human outcomes.

### 8.2 Safety and regulatory considerations

The prebiotics based on polysaccharides are extensively investigated for potential benefits to human health, especially concerning the improvement of gastrointestinal health, modulation of microbiota, improvement of digestion, enhancement of immunity, and management of metabolic diseases such as obesity and diabetes. Nevertheless, despite the potential benefits of prebiotics on human health, the regulations on the use of prebiotics are complex and strictly regulate consumer protection, fair trade practices, and innovation.

The recent rise in the application of Polysaccharides from different natural sources as bioactive food ingredients has led to thorough examination by the United States Food and Drug Administration (US-FDA) and the European Food Safety Authority (EFSA) [312]. These organizations regulate claims on health concerning natural polymers to ensure that the available scientific evidence supports the claims made on these polymers. Moreover, national laws and standards set by the Codex Alimentarius Commission (CODEX) have to be met to allow the application of these polymers in food products [313].

Despite thorough investigation of the functional properties of these polymers, very few have been approved for application in food products. This can be attributed to factors such as insufficient sample sizes used in research studies, difficulties in mimicking functionality in vitro, *in vivo*, and in humans, as well as the absence of thorough studies on the potential toxicity of these polymers. To support prebiotic claims, there should be evidence of the prebiotic activity at different levels of research studies, including laboratory research, animal studies, human studies, and epidemiological studies [314]. According to global laws on the regulation of function or health claims on prebiotics, these claims should be supported by thorough scientific examination based on universally accepted standards [315].

Evidence from polysaccharides and gut microbiome research indicates that most natural Polysaccharides are of low toxicity and possess considerable bioactive potential for food and pharmacological uses [4,5,312,313,316]. However, several limitations need to be overcome to enable the effective and safe use of Polysaccharides. Since most prebiotic activity is confirmed through fecal fermentation assays, proper sample collection, handling, and analysis of Polysaccharides and fecal samples are essential.

For better reproducibility of polysaccharides-fermentation assays, future research should consider the implementation of standardized in vitro fermentation assays that include: (i) physicochemical analysis of Polysaccharides (e.g., molecular weight distribution, linkage composition, and degree of branching), (ii) fecal donor criteria and dietary background and health conditions, (iii) substrate dosage ranges, (iv) fermentation duration and anaerobic conditions, and (v) endpoints such as microbial composition (16S rRNA and shotgun metagenome sequencing), SCFAs production, and pH measurement. The integration of metagenome and metabolome analysis would enable the identification of specific carbohydrate enzyme activity and metabolite production, leading to a better understanding of the fermentation process.

Though clinical trials have consistently found beneficial modulation of the gut microbiota and SCFAs production with the supplementation of polysaccharides, there is considerable variation in the extent of the effect. This variation can be attributed to the differences between the microbiota of each individual, the polysaccharide structure, the dose of the polysaccharide, etc. Thus, it can be noted that the inconsistencies found do not detract from the potential of polysaccharides, but it emphasizes the importance of standardizing the structure of the polysaccharide, the diet, etc. Thus, it is evident that research should be conducted to understand the effect of the variations of the microbiome of each individual with the consumption of polysaccharides, leading to the formulation of a personalized diet. Further hypothesis-driven clinical research is needed to understand the cause-and-effect relationship between the modulation of the gut microbiome and the health of the body.

Although the prebiotic activities of many Polysaccharides have been proven in the clinical setting, the effectiveness of Polysaccharides may vary depending on the individual's microbiota composition, dosage, and delivery form [4,19,25,27,177]. Furthermore, the long-term safety of Polysaccharides, particularly at high doses, is still under investigation. It is important to conduct long-term studies to ensure the long-term safety of Polysaccharides, as well as their compliance with regulations, while avoiding possible interactions with other food components or medications [316].

## 9 Future perspectives

Despite these immense potentials, there are various limitations to Polysaccharides. One of these limitations is the interaction between Polysaccharides and gut microbiota. The lack of standardized approaches in characterizing gut microbiota is a major limitation in understanding polysaccharides-gut microbiota interactions. The identification of specific polysaccharides that display scientifically validated prebiotic activities and optimizing their structural features to maximize their functionality are also challenges in Polysaccharides.

Another limitation in the validation of Polysaccharides is the lack of large-scale, well-conducted clinical trials. It is difficult to translate results from animal and in vitro models to human applications. The complex regulatory environment in polysaccharides-based products is also a limitation, particularly in relation to supplement quality control and standardization.

In terms of future perspectives, there are various opportunities to overcome these challenges. The development of in silico models and multi-omics approaches will greatly help in understanding polysaccharides-gut microbiota interactions. Another potential opportunity is personalized nutrition, which can greatly contribute to polysaccharides-based therapy. It can also greatly help in advancing personalized medicine. Polysaccharides can also greatly contribute to emerging areas such as gut-brain axis, neurological diseases, and immune system regulation. To maximize these potentials, there is a need to develop sustainable approaches to large-scale production of microbial Polysaccharides.

Moreover, future research should aim to transcend descriptive microbiota changes and adopt operational approaches to deliver personalized polysaccharides-based interventions. A logical approach to this would be to incorporate functional microbiome characterization, such as shotgun metagenome sequencing for CAZyme content and polysaccharides utilization loci (PULs), to classify individuals according to their polysaccharides degradation potential. This should then be followed by a series of structure-based interventions, where Polysaccharides are comprehensively characterized for linkage patterns and molecular weight distribution, and then linked to metabolomics to correlate structural characteristics to specific metabolic outcomes, including SCFAs, bile acids, and indoles. A longitudinal study design, including host metabolome and inflammation markers, is also essential to differentiate between temporary fermentation responses and lasting microbiome remodulation. Additionally, intervention studies on the effects of processing-induced structural variations and food matrix incorporation on polysaccharides fermentability and microbiome functionality are also essential to bridge mechanistic research to practical applications for polysaccharides-based nutrition.

## CRediT authorship contribution statement

**Athira Jayasree Subhash:** Data curation, Visualization, Writing – original draft, Writing – review & editing. **Mohamed Abdin:** Visualization, Writing – original draft, Writing – review & editing. **Gafar Babatunde Bamigbade:** Visualization, Writing – original draft, Writing – review & editing. **Maduni Paththuwe Arachchi:** Visualization, Writing – original draft, Writing – review & editing. **Naeem Ullah:** Writing – original draft, Writing – review & editing. **Mutamed Ayyash:** Conceptualization, Funding acquisition, Project administration, Resources, Supervision, Writing – original draft, Writing – review & editing.

## Declaration of competing interest

The authors declare that they have no known competing financial interests or personal relationships that could have appeared to influence the work reported in this paper.

## Data Availability

No data was used for the research described in the article.

## List of abbreviations

AG (Acacia Gum); ALT (Alanine Aminotransferase); ATPE (Two-Phase Aqueous Extraction); BGE (β-*d*-Glucan-Enriched); BSGLP (Polysaccharides from Sporoderm-Broken Spores of *Ganoderma lucidum*); CAZymes (Carbohydrate-Active Enzymes); CGN (Carrageenan); CODEX (Codex Alimentarius Commission); DE (Degree of Esterification); DES (Deep Eutectic Solvents); DSG (Deshipu Stachyose Granules); EFSA (European Food Safety Authority); F/B (Firmicutes to Bacteroidetes Ratio); FCPS (*Ficus carica* Linn. Polysaccharide); FCPG (*Ficus carica* Linn. and Peach Gum Polysaccharide Complex); FXR (Farnesoid X Receptor); GAGs (Glycosaminoglycans); GC (Gas Chromatography); GPC (Gel-Permeation Chromatography); HAOS (Heterozygous Alginate Oligosaccharides); HCD (High Cellulose Diet); HFD (High-Fat Diet); HHGG (High Molecular Weight Partially Hydrolyzed Guar Gum); HPLC (High-Performance Liquid Chromatography); HPGG (Partially Hydrolyzed Guar Gum); KO3 (κ-Carrageenan Oligosaccharides with Smaller Degree of Polymerization); KO6 (κ-Carrageenan Oligosaccharides with Larger Degree of Polymerization); LCD (Low Cellulose Diet); LBG (Locust Bean Gum); LBGH (Locust Bean Gum Hydrolysate); LBP (*Lycium barbarum* Polysaccharide); LW-XG (Low Molecular Weight Xanthan Gum); MAE (Microwave-Assisted Extraction); MAOS (Mannuronic Acid Oligosaccharides); MHGG (Medium Molecular Weight Partially Hydrolyzed Guar Gum); MOS (Manno-Oligosaccharides); MS (Mass Spectrometry); NAFLD (Non-Alcoholic Fatty Liver Disease); NCC (Nano-Crystalline Cellulose); NMR (Nuclear Magnetic Resonance); PGR (Policaptil Gel Retard®); PHGG (Partially Hydrolyzed Guar Gum); PLP (Polysaccharides from Phellinus linteus); PMP (1-Phenyl-3-Methyl-5-Pyrazolone); RS (Resistant Starch); SA (Sodium Alginate); SCFAs (Short-Chain Fatty Acids); SXRG84 (Xylorhamnoglucuronan from Ulva sp. 84); SWE (Subcritical Water Extraction); T2DM (Type 2 Diabetes Mellitus); UAE (Ultrasound-Assisted Extraction); UMAE (Ultrasound-Microwave-Assisted Extraction); UPAE (Ultrahigh Pressure-Assisted Extraction); US-FDA (United States Food and Drug Administration); ULP (Ulva lactuca Polysaccharide).
